# Closed to reason: time for accountability for the International Narcotic Control Board

**DOI:** 10.1186/1477-7517-4-13

**Published:** 2007-05-08

**Authors:** Dan Small, Ernest Drucker

**Affiliations:** 1 PHS Community Services Society, Vancouver, Canada; 2 Department of Anthropology and Sociology, University of British Columbia, Vancouver, Canada; 3 Department of Medicine, University of British Columbia, Vancouver, Canada; 4 Montefiore Medical Center, Albert Einstein College of Medicine, Bronx, NY, USA

## Abstract

For more than two decades, the International Narcotic Control Board (INCB) has tried to stop harm reduction and its HIV prevention programs. This posture is based on a fundamental misunderstanding of their responsibilities and of drug addiction itself – i.e. as a public health and clinical care matter made criminal by decree. A recent focal point for the Board's action has been rejecting the use of supervised injection facilities to reduce morbidity and mortality of drug injectors. They single out individual countries and attempt to bully them into rejecting such programs under the banner of the United Nations (falsely) and in the name of international treaties. Their unrelenting and unjustified badgering of signatories to the international treaties that established the INCB is not only unjustified; it is an affront to one of the core purposes of the Board itself: to ensure adequate medical supplies and safe use of controlled substances. The INCB's ill-conceived obsession with intravenousaddiction as a crime flies in the face of the medical view and policies of the World Health Organization and the universally endorsed principles of the General Assembly of the United Nations.

The latest target of the INCB is North America's only supervised injection facility, *Insite*, located in the inner city of Vancouver, Canada. Using the power of their office to meddle in matters of public health for individual nations is without medical, scientific or legal justification. But, most importantly, it is a matter of lifeand death for these most marginalized of citizens. The empirical evidence remains that a significant portion of the continued growth of the AIDS pandemic is due to injecting drug use, and the INCB's intrusion will inevitably result in additional deaths due to preventable HIV infections and drug overdoses.

So we are very pleased to call to our readers' attention to a recent report produced by the Canadian HIV/AIDS Legal Network and the International Harm Reduction Development Program (IHRD) joined by former United Nations Special Envoy for HIV/AIDS in Africa, the respected Canadian statesman Stephen Lewis. The full report, *"Closed to Reason: The International Narcotics Control Board and HIV/AIDS" *is attached along [see Additional file 1] with a Russian translation of the key findings of the authors [see Additional files 2] as well as Russian and Chinese translations of this abstract [see Additional 3 and 4]. As the report makes very clear, the time to inject some accountability and reason into the INCB is now.

Howmany times must a man look up

Before he can see the sky

Yes and how many ears

Must one man have

Before he can hear people cry?

Yes, and how many deaths

Will it take till he knows

That too many people have died?

Bob Dylan

## Background: facing the epidemic

It has been over 25 years since physician Michael Gottlieb identified the first clinical cases of AIDS in the San Francisco [1]. But AIDS continues to ravage the world. More than 25 million people nearly the population of Canada have died of AIDS, 15 million children have been orphaned by AIDS and 65 million people having been infected with the preventable HIV virus[2] AIDS is now the leading cause of death in the world for people aged 15 to 49 years of age[3] A central feature of the last two decades of the AIDS pandemic has been iintravenous drug use – which continues to be responsible for a massive wave of new HIV infections worldwide[4] Harm Reduction programs, that provide sterile syringes, Supervised Injection Facilities, and innovative overdose prevention programs that help bring users to drug treatment and HIV screening and treatment have now laid the foundation for reducing the spread of AIDS in this population.

While the INCB has, after 20 years of opposition and obstruction of harm reduction measures, now (grudgingly) recognized that needle exchange and substitution treatment are not in conflict with the international conventions under which the INCB established. But the INCB continues, in effect, to demonize needle distribution (and harm reduction measures in general) by portraying such measures as being at perpetual risk of contradicting the sacred goal of demand reduction. Therefore, needle distribution programs; if they are to exist at all, must be purged of any sign of "encouraging" drug use. It is telling that the INCB concludes its description of needle distribution with the following proclamation:

"The Board welcomes the United States Government's unequivocal policy position against any form of legalization of the non-medical use of drugs". [5] (p. 10).

By linking what they call "needle distribution schemes" to drug "legalization", the INCB constructs an ideological barrier to HR programs. Rather than act as an aid in the establishment of widespread needle distribution programs, the INCB continues to undermine them. And if the INCB notes that " governments may, lawfully, implement measures such as needle distribution programs to reduce the spread of HIV/AIDS" it then states, with inherent contradiction typical of its pronouncements, that any "prophylactic measures should not promote and/or facilitate drug abuse" [6] (p. 1). Implicit in this declaration is the assumption that somehow harm reduction measures might promote or facilitate drug abuse – for which there is not a shred of justification, or of evidence. This position prefigures (and explains) their attack on Supervised Injection Facilities:

""The Board has stated on a number of occasions, including its recent Annual Reports, that the operation of such facilities remains a source of grave concern. The Board reiterates that they violate the provisions of the international drug control conventions."

"The Board reiterates that article 4 of the 1961 Convention obliges States to ensure that the production, manufacture, import, export, distribution of, trade in, use and possession of drugs is to be limited exclusively to medical and scientific purposes. Therefore, from a legal point of view, such facilities violate the international drug control conventions."

So, then, the INCB appears to begrudgingly allow for needle distribution programs, as long as injecting drug users, many of whom are the most marginalized of citizens, inject drugs furtively and outside of a healthcare setting designed to prevent overdoses and HIV infections.

In British Columbia, needle distribution has historically been similarly considered as closely connected to a demand reduction strategy. This meant a kind of "one for one" policy whereby needles were not be given unless injection drug users provided a needle in exchange. This strategy was aimed, in part, at ensuring that people with active addictions satisfactorily disposed of their needles. As well, there was a limit (e.g. six per day) placed on the number of needles that were allowed per injection drug users. This limit was regardless of the persons injecting practices or drug of choice (cocaine users would inject many times per day versus heroin users who would inject less frequently for example). The placing of a limit on the number of syringes allowed embodied a kind of "demand reduction" value inherent in the delivery of the service. This meant that when people required many needles for drug use, they were limited at 6 per day regardless of their need. The needle exchange at that time was a centralized rather than a decentralized. There are those that believe that this implicit "demand reduction" approach to needle "exchange" rather than "distribution" (unlimited syringe access) contributed, in part, to the explosion of HIV and HCV in the IDU population in the region.

While the INCB overtly concludes that the NEP are technically allowed this does not mean that the Board, in actuality, entirely supportive of this harm reduction measure. Their stance on Supervised Injection Facilities is indicative of their "true colors" on this life saving matter. These kinds of mediocre stands when it comes to life saving programs like NEP have drastic consequences, on the ground, for marginal IDU populations where local governments struggle to address the AIDS pandemic. What they require is support and leadership, not mixed messages from an international body invested with the prestige of the global community.

When it comes to the pandemic of AIDS and Injection Drug Use, what is required is clear leadership with respect to strategies that will save lives. The faint and clarified praise for NEP and rejection of SIFs by INCB is a barrier to implementing critical population health initiatives. The Board's implicit and explicit resistance to harm reduction is especially influential in the minefield of morality within which injection drug use and AIDS are situated. The politics of blame surrounding people living with active addiction make the conditional support of the INCB especially unhelpful and even more damning.

In times of sweeping death due to uncontrolled illness, an unsure healthcare intervention is better than no medical intervention at all. Better yet, a healthcare intervention with clear efficacy and evidence base, such as SIFs, even though culturally controversial, demand action rather than clarified and conditional support. Clarified supports are the kind where a population healthcare intervention like a SIS might be possible an "ideal world", when the "trees all grow to heaven" and the populace is blessed with a month of Sundays with eternal sunshine. SIFs cannot wait for this month of Sundays. Their time is now and by opposing their establishment, the INCB is being a part of the problem rather than the solution for the AIDS pandemic.

Supervised injection facilities (SIF) are, to a large extent, an HIV prevention initiative that provides clean injection equipment. Vancouver's SIF, Insite, supervises approximately 20,000 injections every month. Every one of these 20,000 occurrences is an injection that is not shared and makes use of a clean syringe. To a large extent, the SIF is a needle distribution program and, for this key feature, as well as its allowance of injection itself under supervised conditions, it has come under condemnatory fire from the International Narcotic Control Board. Of course, everyone, including both the operators and users of the facility, hopes for abstinence and restoration to full health, but for now, the prevention of HIV, HCV and fatal overdoses is a good medical beginning. In medicine, a partial medical intervention, with efficacious outcomes, is better than no intervention at all. Sometimes, rather than shooting for the stars such as eradicating addiction altogether, we have to satisfy ourselves with more earthly outcomes such as preventing an HIV infection.

Needle distribution is a hallmark component of an SIF. The efficacy of needle distribution is no longer open for debate in the medical and scientific community. The World Health Organization (WHO) has reviewed the available scientific and medical literature on harm reduction programs and concluded that the evidence overwhelmingly indicates that providing such services for injecting drug users significantly reduces HIV infections:

"The studies reviewed in this report present a compelling case that NSPs substantially and cost effectively reduce the spread of HIV among IDUs and do so without evidence of exacerbating injecting drug use at either the individual or societal level. This suggests that authorities responsible for areas threatened by or experiencing an epidemic of HIV infection among IDUs should adopt measures urgently to increase the availability and utilization of sterile injecting equipment and expand implementation to scale as soon as possible. conditions and other approaches better suited in other places and conditions. The important point is to aim to reduce the circulation time of needles and syringes."[7] (p. 30)

In recognition of the need for efficacious and evidenced-based action with respect to the AIDS pandemic, the United Nations General Assembly unanimously adopted an imperative Resolution to address the AIDS on 2 June 2006. In this resolution, the United Nations General Assembly **unanimously **publicly declared the importance of harm reduction and needle distribution by reiterating that:

"...prevention of HIV infection must be the mainstay of national, regional and international responses to the pandemic, and therefore commit ourselves to intensifying efforts to ensure that a wide range of prevention programmes that take account of local circumstances, ethics and cultural values is available in all countries, particularly the most affected countries, including information, education and communication, in languages most understood by communities and respectful of cultures, aimed at reducing risk-taking behaviours and encouraging responsible sexual behaviour, including abstinence and fidelity; expanded access to essential commodities, including male and female condoms and **sterile injecting equipment; harm-reduction efforts related to drug use; **expanded access to voluntary and confidential counselling and testing; safe blood supplies; and early and effective treatment of sexually transmitted infections;" [emphasis added][2] (p. 4)

And yet, despite the overwhelming evidence and consensus of professional judgment support the importance of harm reduction (including needle distribution and the healthcare centres from which clean injection equipment is distributed, supervised injection facilities) another international organization, the International Narcotic Control Board (INCB), has been publicly attacking these health programs. The INCB gains its authority from three treaties, the Single Convention on Narcotic Drugs (1961), the Convention on Psychotropic Substances (1971) and the United Nations Convention against Illicit Traffic in Narcotic Drugs and Psychotropic Substances (1988)[8] The INCB, generally speaking, is responsible for ensuring that adequate supplies of narcotic drugs are available in the world for medical and scientific use while, conversely, pointing to weak points in the controls of these same substances that lead to the sale, use or manufacturing of illicit drugs. The INCB monitors governments with respect to their control of illicit drugs and makes recommendations to assist each signatory to the aforementioned treaties to maintain their nation's responsibility with respect to the distribution of medically sanctioned drugs and the corresponding prevention of illicit drugs. The INCB is primarily concerned with the international and national flow of drugs, both illicit and licit.

## Unlucky number 13: the 13 member board of the INCB assail HIV prevention

Incongruously, the INCB has become confused about HIV prevention programs and, as a result, developed a pattern in recent years of speaking out against accepted medical and public health strategies. The 13 person committee governing of the INCB, often mistakenly understood by the public to be synonymous with the United Nations, seems to have decided that it is not accountable to scientific evidence, the opinion of most public health officials, nor to the mounting toll of AIDS due to syringe sharing among injecting drug users. As a result, the INCB is beginning to play a central role in promoting unsafe injection practices (by ruling out syringe distribution as a healthcare initiative), and, inadvertently, spreading regional AIDS outbreaks.

In marked contrast to the vast majority of the medical and scientific establishment, the INCB sees SIFs as neither medical nor scientific and this is basis of their repeated public grievances about purported infringements on international drug control treaties. In so doing, the board is continuing to chase its imaginary tail with increasing fervor within an unaccountable and unscientific universe of its own construction. This would be allowable if AIDS were not such a serious matter.

By showing aggression towards SIFs, the International Narcotics Control Board is undermining HIV prevention initiatives that save lives. The INCB began declaring that SIFs are in breach of international treaties in 2001 and 2002 when they stated:

"Establishing drug injection rooms, where drug abusers can inject drugs that they have acquired from illicit sources, is contrary to the international drug control treaties"(p. 70)[9].

Since that time, the INCB has repetitively attempted to undermine Vancouver's SIF, Insite, the only such health program in North America. In 2003, from their headquarters in Vienna, the INCB proclaimed that Insite violates international law and attempted to urge the Canadian government to comply with their wishes that the program be closed[10] In 2004, the INCB singled out Australia's lone SIF as an affront to international drug control treaties[11] Similarly in 2005, the INCB chastised Norway for establishing a SIF and stated that injection rooms "facilitate" the use of drugs[12] Once again, the Board misunderstood the deeply medical purpose of these healthcare initiatives, that is, to avert wherever possible needless deaths due to drug addiction due to preventable overdoses and HIV and HCV infections. In 2006, the INCB turned its attention to Switzerland and highlighted the addition of "inhalation" components of this country's multifaceted consumption rooms as unlawful. Likewise, the board paid a visit to Germany, chastised this country for their harm reduction efforts in operating SIFs. They then lamented the existence of such medical initiatives Switzerland, Canada, Luxembourg, the Netherlands, Norway, Spain and Australia[13] Finally, the group mounted its most recent attack in the press against Vancouver's the life-saving injection facility in March of 2007 and vowed to pressure the Canadian government directly to have the medical program shut[14].

In fact, the international treaty to which the INCB repeated refer is the Single Convention on Narcotics of 1961 (amended in 1972). The Preamble of the Convention begins by highlighting that the signatories are primarily concerned with the wellbeing of humanity and main purpose of the convention is to relieve suffering:

"The Parties, concerned with the health and welfare of mankind, recognizing that the medical use of narcotic drugs continues to be indispensable for the relief of pain and suffering and that adequate provision must be made to ensure the availability of narcotic drugs for such purposes" (p. 1)[15].

The ensuing section trumpets about the importance of being committed to fighting the forces of evil, language that was indicative of the spirit of the time half a century ago when the agreement was drafted, before highlighting the importance of the drugs in the context of medical and scientific use. In fact, it is precisely for scientific or medical purposes that SIFs are eligible for exemption under Section 56 of the *Controlled Drug and Substances Act *(CDSA) of Canada.

AIDS is a public problem for all of humanity. Similar to when a situation or area is designated as a national disaster, the categorization of an epidemic brings public attention as well as national resources. When a disease outbreak is categorized as an epidemic, it also goes through a social transformation to become a public problem. Not all social problems are declared public problems [16]. For example, homelessness might be considered a public problem in some areas but not in others. When a problem becomes a public problem then the government officially has to take on responsibility to address it. Mental illness, poverty and drunken driving have not in the past been considered public problems, whereas today they are expected to be focus of government attention and the responsibility of officials and publicly funded agencies. Drug addiction, as a leading cause of HIV infection, is a public problem.

Certain words are highly significant, like the word pandemic, and they are moored in significant places in the social space and only certain people are consecrated with the social privilege to employ them [17]. Just as only a physician can pronounce someone dead, only certain medical professionals are consecrated with the authority to declare an epidemic (such as, in today's context, the Chief Medical Health Officer, the Health Minister, the Health Board). Similarly, these same socially designated bodies can socially bury or put out of sight problems that require action. The United Nations is such a body; it can, through an act of social magic, raise a problem to the national stage or, through inaction, relegate the same problem to the shadows.

Only certain societal bodies or individuals such as teachers, priests, doctors or legislators are delegated with the authority to carry out key responsibilities [17]. For example, the christening of a yacht can only be completed by a designated person can perform the task of christening. Similarly, at the College of Physicians and Surgeons, only the President can deliver a reprimand to a physician guilty of professional violation. It would socially awkward indeed if a passerby were to spontaneously break a bottle of champagne on the side of a boat at a christening ceremony or if a public member of the College of Physicians Council proceeded to take the lead in reprimanding a physician. In certain social settings, only the designated representative has been invested with social authority to perform significant tasks. In the case of the pandemic of AIDS, the socially designated institutions responsible for fighting HIV are many including the United Nations, the World Health Organization, each sovereign country and, perhaps, all of us.

Key societal institutions, such as the INCB, have the social authority to consecrate certain states of affairs:

"To institute, in this case, is to consecrate, that is, to sanction and sanctify a particular state of things, an established order, in exactly the same way that a *constitution *does in the legal and political sense of the term. An *investiture *(of a knight, Deputy, President of the Republic, etc.) consists of sanctioning and sanctifying a difference (pre-existent or not) by making it *known *and *recognized*; it consists of making it exist as a social difference, known and recognized by the agent invested and everyone else" [17: 119].

The institution achieves a kind of social wizardry by anointing the credentials of key individuals, such as Chief Medical Health Officers, Surgeon Generals or Chief Coroners, who have the official qualifications to declare public problems such as epidemics that demand widespread attention and resources.

With respect to the historical analysis of epidemics, it is often the case that what was not studied is as revealing as what is studied [18]. With respect to AIDS, there was lengthy period of time in the United States during which the actual acronym was never mentioned publicly or in any official correspondence of the President. AIDS was, officially, culturally erased. As a result, national resources could not be dedicated to addressing an important issue that, for all intents and purposes, did not exist overtly.

## The time for reason

So we are very pleased to call to our readers attention to a recent report produced by the *Canadian HIV/AIDS Legal Network *and the *International Harm Reduction Development Program (IHRD)*, joined by former United Nations Special Envoy for HIV/AIDS in Africa, Stephen Lewis, who held a press conference to release ***"Closed to Reason: The International Narcotics Control Board and HIV/AIDS."***[19] The full report is attached. [20]

The new report details the ways in which the INCB, funded and staffed by the UN, has "blocked effective HIV prevention among drug users."[19] The document focuses on errors of fact and omissions in INCB publications and statements, the ways in which the Board has ignored expert legal counsel and scientific evidence, and the need for greater accountability and transparency at the INCB. The reports **recommendations are clear and compelling**.

"To improve accountability, address the HIV epidemic, and meet its mandate to assess compliance with the UN drug conventions, the INCB must change:

• The INCB should regularly assess the supply and adequacy of treatment for chemical dependence. It should provide technical assistance to help countries accurately estimate the need for opiate substitution treatment, support governments that are striving to scale up such treatment, and encourage governments that have yet to provide these life-saving therapies to find safe and effective ways to do so.

• The INCB should cite scientific evidence for its observations about drug use and health, and legal grounds for its interpretation of law. It should provide sources of information for its annual reports, and opportunities for UN member states and civil society groups to offer corrections or additional information.

• The INCB should provide greater opportunity for exchange with UN member states, UN agencies with relevant mandates, civil society, and HIV/AIDS experts. INCB country missions should include greater opportunities for engagement with these groups.

• The World Health Organization, the UN Economic and Social Council (ECOSOC) and UN member states should ensure that INCB members include persons with expertise in HIV/AIDS policy and international law.

• The INCB should articulate, and ECOSOC should evaluate, public guidelines to clarify when INCB members are speaking for the Board, and how misstatements of fact can be corrected.

• The UN Secretary-General should commission an independent evaluation of the INCB, including a scientific evaluation of the Board's statements on health, and an examination of Board members' independence and expertise, with particular attention to HIV, international law and human rights"(pp. 3–4)[20].

## Conclusion

If the INCB were to be successful in just one of their foolhardy attempts to bully governments into closing a single SIF as a needle distribution centre, then this organization would, quite simply, have blood on its hands. Which of these 13 people, self-righteously sitting on the INCB, would be willing to face the consequences of their having sacrificed medicine and science on the alter of a 46 year old treaty about which even their legal interpretation is uncertain? Or, which of them, if any, would willing to face the father or mother of the addict who died of a drug overdose in an alleyway? Would they proudly talk of their successful mission from their comfortable office in Vienna to an injection room that they had, through their lobbying efforts, successfully closed down?

The Preamble of the Charter of the United Nations as enshrined since 1945, states the peoples of the UN are committed to work towards:

"... conditions under which justice and respect for the obligations arising from treaties and other sources of international law can be maintained, and to promote social progress and better standards of life in larger freedom, and for these ends, to practice tolerance and live together in peace with one another as good neighbours, and to unite our strength to maintain international peace and security, and to ensure, by the acceptance of principles and the institution of methods, that armed force shall not be used, save in the common interest, and to employ international machinery for the promotion of the economic and social advancement of all peoples..."[15]

Treaties, then, are for the advancement of peoples. Treaties are not to be used to exclude marginalized persons, like injecting drug addicts, from receiving access to basic life saving medical care such as injection facilities and syringe distribution programs. International law is for promoting inclusivity, of everyone, even those with the most comprised social tenure, in the global community.

Three years after the establishment of the UN, in 1948, the General Assembly of the United Nations approved the Universal Declaration of Human Rights. Article 25, under the Declaration, states that every person:

"has the right to a standard of living adequate for the health and well-being of himself and of his family, including food, clothing, housing and medical care and necessary social services, and the right to security in the event of unemployment, sickness, disability, widowhood, old age or other lack of livelihood in circumstances beyond his control" (p. 5)[15].

Every human being, including people living with intravenous drug addictions, has the basic human right to their health and medical care and security of their person. For some people, that inalienable right to health might come through the provision of a clean syringe in an injection facility.

While the increasing implementation of SIF medical initiatives throughout the globe appear to be increasing the travel budget for the INCB and the resultant tourist opportunities of its 13 members as they embark on their "missions", it does not appear to be effective in anyway with respect to increasing their knowledge of population health or epidemiological approaches to healthcare. It appears that the International Narcotics Control Board is unwavering in its steadfast determination to drink its own ideological bathwater in spite of the widespread availability of knowledge available from the wellspring of medicine and science. It is clearly time to retire the International Narcotics Control Board in its current configuration as a cloistered and unaccountable body that is impenetrable to evidence based science and medicine. According to the INCB annual report, the Board reports to the Economic and Social Council of the United Nations. The Economic and Social Council, in turn, has a "reporting, cooperating and advising relationship" with the General Assembly of the United Nations[13].

Every injecting drug addict was once someone's son or daughter. They do not deserve to die from AIDS or drug overdose due to a cloak of ignorance about basic medical evidence that has, for too long, shrouded the International Narcotic Control Board. It is the responsibility of the United Nations to protect their health and well-being.

It is our opinion that the make-up of the governing body of the INCB needs to be dissolved forthwith in its present capacity as a matter of great public urgency in order to reconstitute the board in order to ensure that its make-up is comprised of HIV, medical and legal experts with respect to intravenous drug addiction as a health issue. According to the founding documentation associated with the INCB, the Single Convention on Narcotic Drugs of 1961, the expenses of the Board are ultimately authorized by the United Nations General Assembly[15] In light of this financial reporting relationship, we suggest that the General Assembly of the UN, at the next possible moment, bring to an end any expenditure by the INCB that undermine, investigate (as in flight travel to visit and badger) or publicly criticize accepted HIV prevention programs including SIFs. What's more, we call on the United Nations to immediately put in place the legal means to repeal the 1961 Single Convention on Narcotic Drugs in order to replace it with an updated language that reflects the fact that serious drug addiction is a medical issue and not a crime as a "harm reduction measure" to prevent subsequent generations of INCB members from being similarly confused as the present participants on the governing committee.

The time for accountability for the INCB is now. We are calling on the general assembly of the United Nations, the signatory nations to the treaties that created the mandate for the INCB, to take steps to contain the damage done by stopping the deleterious work of the INCB before more injecting drug users needlessly die of AIDS and drug overdoses because of the ill-conceived and obstinately counterproductive efforts of this agency. We cannot allow this public body to continue to function in a way so disconnected from the medical and scientific evidence about drug use and AIDS. In the face of the almost universal condemnation of the INCB by the legal community, the INCB's continued arrogance and intransigence sullies the reputation of the United Nations and violates the humanitarian spirit in which this board and all UN agencies were created. The goals of INCB in this matter should be congruent with those of the United Nations and World Health Organization: to promote rather than hinder public health and, in so doing, save lives – not to bring about unnecessary deaths due to preventable HIV infections and AIDS.

## Supplementary Material

Additional file 1 Closed to Reason: The International Narcotics Control Board and HIV/AIDS. Report by J. Csete and D. Wolfe of the Canadian HIV/AIDS Legal Network; International Harm Reduction Development Program (IHRD); Open Society Institute (OSI); 2007:1–32. Click here for file

Additional file 2 Closed to Reason: The International Narcotics Control Board and HIV/AIDS Key Findings. Russian translation of key findings of the above report. Click here for file

Additional file 3 *"Closed to Reason": Time for Accountability for the International Narcotic Control Board*. Russian translation of abstract of the above editorial. Click here for file

Additional file 4 *"Closed to Reason": Time for accountability for the International Narcotic Control Board*. Chinese translation of abstract of the above editorial. Click here for file
