# Supplementary material for: Closed to reason: time for accountability for the International Narcotic Control Board
Source: Harm Reduct J. 2007 May 8;4:13. doi: 10.1186/1477-7517-4-13 (PMC1871577; doi:10.1186/1477-7517-4-13)
Supplement: Additional file 1 — Closed to Reason: The International Narcotics Control Board and HIV/AIDS. Report by J. Csete and D. Wolfe of the Canadian HIV/AIDS Legal Network; International Harm Reduction Development Program (IHRD); Open Society Institute (OSI); 2007:1–32. [file 1477-7517-4-13-S1.pdf]

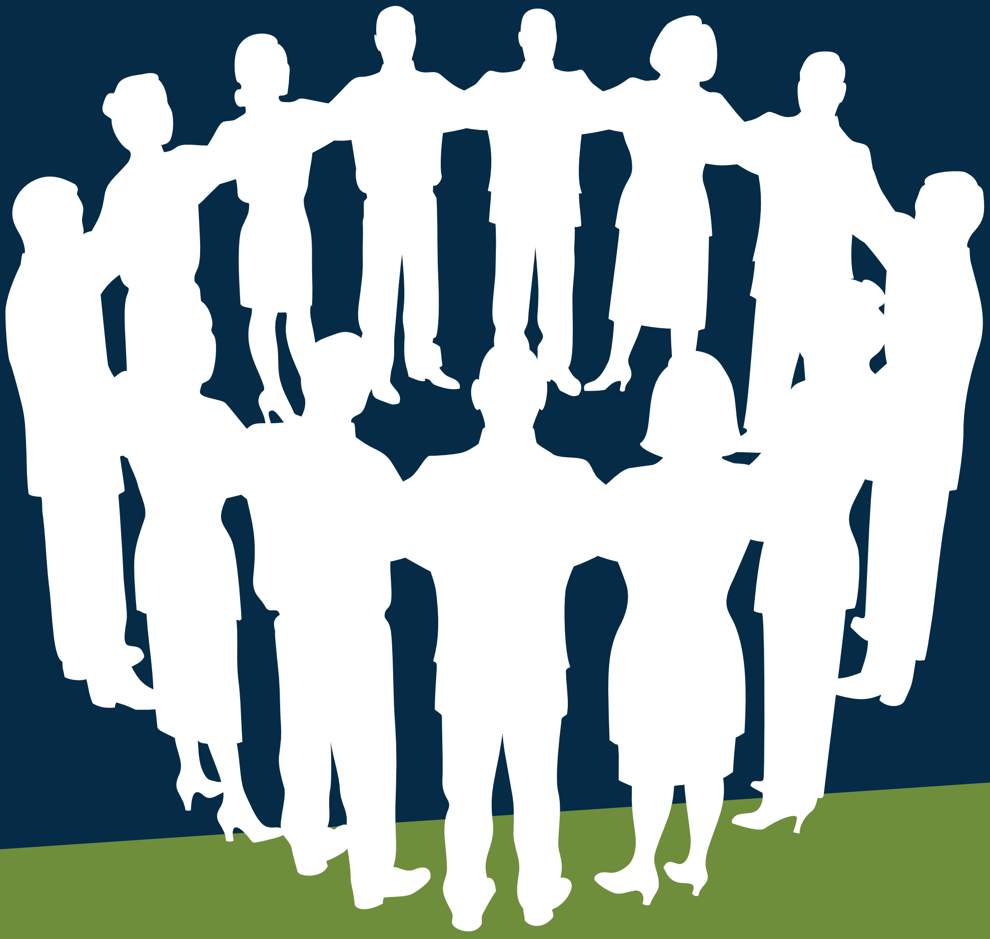

Closed to Reason:

The International Narcotics Control Board and HIV/AIDS

Canadian HIV/AIDS Legal Network
Réseau juridique canadien VIH/sida

OPEN SOCIETY INSTITUTE
Public Health Program

Canadian HIV/AIDS Legal Network | Réseau juridique canadien VIH/sida

OPEN SOCIETY INSTITUTE
Public Health Program

Closed to Reason:
The International Narcotics Control Board and HIV/AIDS

Canadian HIV/AIDS Legal Network and
International Harm Reduction Development Program (IHRD) of the Open Society Institute
February 2007

Closed to Reason:
The International Narcotics Control Board and HIV/AIDS

Joanne Csete and Daniel Wolfe

© 2007 Canadian HIV/AIDS Legal Network and Open Society Institute

Further copies can be retrieved at www.aidslaw.ca/drugpolicy or at www.soros.org/harm-reduction.

Cataloguing in publication data

Csete J and D Wolfe (2007). Closed to Reason: The International Narcotics Control Board and HIV/AIDS. Toronto/New York: Canadian HIV/AIDS Legal Network and the International Harm Reduction Development (IHRD) Program of the Open Society Institute.

ISBN 978-1-896735-82-5

Acknowledgements
The authors are grateful to the following persons for research assistance and helpful comments on drafts of this report: Taslim Madhani, Richard Elliott, Leon Mar and Richard Pearshouse of the Canadian HIV/AIDS Legal Network, and Kasia Malinowska-Sempruch, Jonathan Cohen, Sophie Pinkham and Elizabeth Eagen of the Open Society Institute. Ari Korpivaara and Will Kramer of the Open Society Institute provided editorial assistance, and Vajdon Sohaili and Leon Mar of the Legal Network oversaw layout and design.

Illustration by Conny Schwindel

About the Canadian HIV/AIDS Legal Network
The Canadian HIV/AIDS Legal Network (www.aidslaw.ca) promotes the human rights of people living with and vulnerable to HIV/AIDS, in Canada and internationally, through research, legal and policy analysis, education, and community mobilization. The Legal Network is Canada's leading advocacy organization working on the legal and human rights issues raised by HIV/AIDS.

About the International Harm Reduction Development Program (IHRD)
The International Harm Reduction Development Program (IHRD), part of the Public Health Program of the Open Society Institute (OSI), works to reduce HIV and other harms related to injecting drug use and to press for policies that reduce stigmatization of illicit drug users and protect their human rights. IHRD, which has supported more than 200 programs in Central and Eastern Europe, the former Soviet Union, and Asia, bases its activities on the philosophy that people unable or unwilling to abstain from drug use can make positive changes to protect their health and that of their families and communities. Since 2001, IHRD has prioritized advocacy to expand availability of needle exchange, opiate substitution treatment, and treatment for HIV; to reform discriminatory policies and practices; and to increase the political participation of people who use drugs and those living with HIV.

About the Open Society Institute (OSI)
The Open Society Institute (OSI) works to build vibrant and tolerant democracies whose governments are accountable to their citizens. To achieve its mission, OSI seeks to shape public policies that assure greater fairness in political, legal, and economic systems and safeguard fundamental rights. On a local level, OSI implements a range of initiatives to advance justice, education, public health, and independent media. At the same time, OSI builds alliances across borders and continents on issues such as corruption and freedom of information. OSI places a high priority on protecting and improving the lives of marginalized people and communities.

Table of Contents

Key findings and recommendations 1

I. Introduction 5

II. Role of the INCB 6

A. Expertise on HIV and drug policy 7

III. Statements and positions of the INCB 8

A. INCB and harm reduction 8

Emphasizing fear, downplaying science 8

Ignoring expert legal analysis 9

Promoting opposition to harm reduction in the media 9

B. INCB and opiate substitution treatment 10

Underemphasizing opiate substitution treatment 10

Failing to remark on country advances and failures 10

Stressing drug control at the expense of public health 12

C. INCB and safer injection facilities 12

Condemnation without legal justification 12

Silencing other UN actors 13

D. INCB and sterile syringe programs 13

Misrepresenting law and science 14

E. INCB and the human rights of people who use drugs 14

Silence on abuses at the national level 14

Dissonance with the UN 16

IV. Impartiality, independence and accountability at the INCB 17

Country influence 17

Russia and the case of methadone 17

Influence of the INCB at the UN 18

Lack of public guidelines on attribution of statements or corrections of fact 18

A culture of secrecy 18

V. Conclusions and recommendations 20

Appendix A: INCB and the mechanisms of global drug control 22

Appendix B: The role of the INCB as specified in the UN drug conventions 24

References 26

Key findings and recommendations

The role of drug policy has been transformed since the era in which the International Narcotics Control Board (INCB), the 13-member body responsible for monitoring compliance with the United Nations drug conventions, was conceived. In a time when an estimated 30 percent of infections outside of Africa are due to injection drug use, drug policy is fundamental to the success or failure of the international response to HIV/AIDS. A significant body of scientific evidence has shown the importance of measures such as effective treatment for chemical dependence and provision of sterile syringes in preventing HIV. The INCB, which emphasizes its impartiality, independence and reliance on evidence, has an historic opportunity to help stop the injection-driven HIV epidemics now emerging and exploding in much of the world.

Instead, the Board has become an obstacle to effective programs to prevent and treat HIV and chemical dependence. INCB annual reports are rife with omissions and misrepresentations and lack both scientific documentation and justification for legal opinions. Country visits by INCB representatives fail to highlight law enforcement patterns that accelerate HIV transmission and represent clear human rights violations. Although the Board is responsible for ensuring the global availability of legal opiates, it has not acted to help countries accurately estimate their need for the opiate substitution treatment shown to be effective in reducing HIV risk and increasing adherence to antiretroviral medications.

The Board stresses the drug control aspects of its mandate. Its annual report for 2005, however, refers 18 times to the role of drug use in accelerating HIV transmission in various countries. Tellingly, that report, like other INCB documents, fails to urge countries to pursue proven strategies to reduce HIV transmission among people who inject drugs. While the UN drug conventions clearly mandate treatment for drug users, the INCB has remained silent on the shortage of effective chemical dependence programs, and the abuses committed in the name of drug treatment and rehabilitation.

The United Nations system as a whole is committed to reducing HIV among people who inject drugs, to safeguarding the human rights of people who use drugs, and to increasing accountability and civil society involvement. In this context, the INCB is an anomaly: a closed body, accountable to no one, that focuses on drug control at the expense of public health and that urges national governments to do the same.

INCB members contradict or seek to thwart evidence-based recommendations of other UN bodies and representatives.

- INCB members have spoken out against sterile syringe programs and opiate substitution treatment, despite findings by the Joint United Nations Programme on HIV/AIDS (UNAIDS), the World Health Organization, the United Nations Office on Drugs and Crime (UNODC) and the INCB itself that these measures are effective and important components of HIV prevention. In 2002, the president of the INCB claimed, erroneously, that distribution of sterile syringes contravened the UN drug conventions. In 2005, a memorandum signed by an INCB member from Russia included numerous misstatements of fact meant to discredit the use of methadone as a treatment for opiate addiction.

- The Board has sought to silence UN representatives who support a fuller range of HIV prevention approaches. In 2006, for example, Stephen Lewis, the UN Secretary-General's Special Envoy for HIV/AIDS in Africa, commented favorably on Canadian data showing that a Vancouver safer injection facility had reduced HIV risk. The next day, he received an angry telephone call from the INCB Secretariat and a promise that the Board would write to the Secretary-General to urge that Lewis be censured for support of "opium dens." In that letter, the INCB president expressed disbelief that "any officer of the United Nation [*sic*] could have made such statements," and demanded that Lewis recant.

INCB reports praise governments that violate human rights.

- A Board delegation visited Thailand in 2004, several months after police forces began a "war on drugs" in which human rights experts documented extrajudicial executions, arrest quotas, use of blacklists, and the internment of tens of thousands of people, including many with no history of drug use. In its report issued after the visit, the Board did not condemn the mass arrests; instead, it expressed appreciation for the Thai government's efforts to investigate the killings, despite findings by human rights groups that the government had failed to allow any independent investigations.

- In 2004, after Bulgaria mandated imprisonment for possession of any amount of any illicit drug, fear of arrest caused rates of drug injection and syringe sharing to increase sharply. INCB representatives visited Bulgaria in 2005, but the Board's report made no mention of the harsh drug law or its impact, noting instead that national drug control legislation was "well-developed."

- In Russia, authorities in 2005 moved to reverse a reform that had reduced the numbers imprisoned for very small amounts of drugs. Wholesale and prolonged incarceration had been recognized as contributing to both penitentiary overcrowding and the fact that Russia's HIV epidemic was among the fastest growing in the world. INCB representatives visited Russia during this debate, but the Board's subsequent report made no mention of any discussion of the issue or of concern about the human rights implications of the policy. Instead, the INCB expressed concern about the extent of drug abuse in Russia, and encouraged coordination and cooperation between HIV and drug treatment services.

- Since 1990, China has marked the UN's International Day Against Drug Abuse and Illicit Drug Trafficking with show trials in which drug dealers are sentenced to death, sometimes as crowds chant "kill, kill." Scores have been executed. Despite professed support for sensitive policing, the INCB has failed to criticize either this practice or the police harassment of those seeking to obtain sterile injection equipment, whether in China or in other countries visited by the Board.

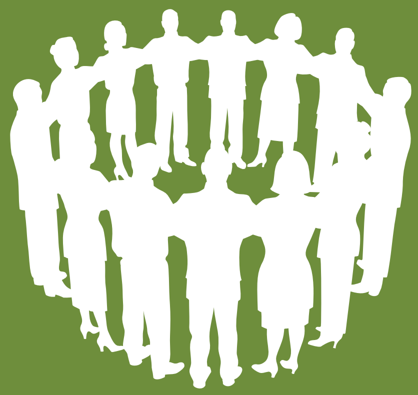

. . . the Board has become an obstacle to effective programs to prevent and treat HIV and chemical dependence.

The Board stresses drug control at the expense of public health, expressing concern about diversion rather than praise for scientifically proven measures that reduce HIV and other harms.

- While acknowledging that WHO added methadone and buprenorphine to its Model List of Essential Medicines in 2005, the Board has made no public effort to promote opiate substitution

treatment (OST) in countries where large numbers of people inject drugs. It also has failed to highlight OST as an essential tool in HIV care or treatment. Although Ukraine, China, Malaysia and Iran have moved in recent years to expand OST programs and/or needle exchange programs to contain HIV, INCB reports have not expressed appreciation for or satisfaction with these developments. Instead, the Board has expressed concern about diversion of methadone and buprenorphine, and urged WHO to advocate for tightened controls on these medications.

The Board issues interpretations of law and pronouncements on harm reduction, despite a lack of expertise in international law and HIV policy.

- According to their published biographies, none of the Board's 13 members has formal training in international law, despite the importance of such credentials in interpreting treaty provisions. In the case of substitution treatment, needle exchange, and safer injection facilities, the pronouncements of INCB members have contravened the findings of the Board's own legal advisors and national experts.

- Despite the centrality of drug use to HIV transmission, none of the Board members has published in peer-reviewed journals on HIV/AIDS, and few list any experience of HIV treatment or prevention in their biographies.

The Board conducts operations in secret, and without mechanisms for accountability.

- INCB meetings are closed to observers, and no minutes are available.

- INCB members have used their Board affiliation when making misstatements of fact, yet no public mechanism exists for member states or community organizations to contest claims, seek clarification, or offer amendments.

- Sources are selectively and inconsistently documented in INCB reports.

- The INCB does not publicize country visits in advance or convene public hearings or other opportunities for input.

- Despite the UN Secretary-General's call for greater transparency and interaction with civil society at the UN, the INCB's website includes no information on the Board's budget or staff. The INCB Secretariat — paid for by the UN — is unresponsive to requests for information from affected communities or non-governmental organizations.

Recommendations

To improve accountability, address the HIV epidemic, and meet its mandate to assess compliance with the UN drug conventions, the INCB must change.

- The INCB should regularly assess the supply and adequacy of treatment for chemical dependence. It should provide technical assistance to help countries accurately estimate the need for opiate substitution treatment, support governments that are striving to scale up such treatment, and encourage governments that have yet to provide these life-saving therapies to find safe and effective ways to do so.

- The INCB should cite scientific evidence for its observations about drug use and health, and legal grounds for its interpretation of law. It should provide sources of information for its annual reports, and opportunities for UN member states and civil society groups to offer corrections or additional information.

- The INCB should provide greater opportunity for exchange with UN member states, UN agencies with relevant mandates, civil society, and HIV/AIDS experts. INCB country missions should include greater opportunities for engagement with these groups.

- The World Health Organization, the UN Economic and Social Council (ECOSOC) and UN member states should ensure that INCB members include persons with expertise in HIV/AIDS policy and international law.
- The INCB should articulate, and ECOSOC should evaluate, public guidelines to clarify when INCB members are speaking for the Board, and how misstatements of fact can be corrected.
- The UN Secretary-General should commission an independent evaluation of the INCB, including a scientific evaluation of the Board’s statements on health, and an examination of Board members’ independence and expertise, with particular attention to HIV, international law and human rights.

I. Introduction

Every year, in late February or March, television and newspaper reports around the world carry headlines such as "UN raps countries over cannabis let-up" or "UN slams drug injection room."[1] These stories come from the annual reports of the International Narcotics Control Board (INCB), a 13-person, ostensibly independent body that does not speak for the United Nations but is an integral part of the UN drug control system.

Like all international drug control bodies, the INCB has a crucial role to play in the global response to HIV/AIDS. HIV is spreading most rapidly in parts of the world where a significant share of HIV infections is linked to illicit drug injection. The Joint United Nations Programme on HIV/AIDS (UNAIDS) estimates that nearly 30 percent of HIV infections outside sub-Saharan Africa are among people who inject drugs.[2] Former Soviet countries that now comprise the Commonwealth of Independent States, much of South and Southeast Asia, and parts of Europe, the Middle East, and Latin America all face injection-driven HIV epidemics. In many of these places, harsh criminal penalties for illicit drug use and social marginalization of people who use drugs impede the implementation and utilization of services for addiction, HIV or other health problems. Since seeking help means risking arrest or social sanction, people who use drugs frequently remain out of reach of HIV prevention services, treatment, or care.

The United Nations system has recognized how central drug policy is to HIV prevention, and has designated the UN Office on Drugs and Crime (UNODC) as the convening agency for all matters pertaining to injection drug use as it relates to HIV/AIDS.[3] Housed at UNODC offices in Vienna and staffed by a 28-member secretariat of UNODC employees, the INCB is one part of an international drug control effort that also includes the Commission on Narcotic Drugs (CND) and the UNODC (see Appendix A).

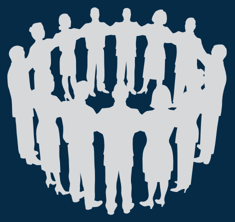

Like all international drug control bodies, the INCB has a crucial role to play in the global response to HIV/AIDS.

This report focuses on the importance of INCB actions to the global response to HIV. Since INCB proceedings are closed to the public, since the Board does not make its letters to governments or minutes from its meetings available, and since the Board has no public procedure for selecting countries to visit or consultations to hold once there, the workings of the Board are difficult to gauge. The findings in this report are based on the review of a variety of materials, including annual and special reports of the INCB, speeches of INCB presidents and members, and other UN documents on HIV and drug policy. Researchers for this report contacted four former and current Board members, as well as the INCB Secretariat, to seek information on questions including the openness of Board proceedings, the criteria by which countries are chosen for INCB visits, and the mechanisms used to ensure Board member independence. No Board member responded. Four months after the initial inquiry, the INCB Secretariat replied with a three-sentence letter recommending that the researchers consult the INCB website. The website provided no information on the questions posed. This lack of transparency at the Board and the reluctance of Secretariat staff to provide information illustrate the need for reform at the INCB.

II. Role of the INCB

The INCB is responsible for monitoring the implementation of three international drug control treaties known collectively as "the UN drug conventions."[4] These conventions oblige governments to curb the illicit supply, traffic and consumption of narcotic and psychotropic drugs, while making such drugs available for medical purposes. (For more information on the drug conventions, see Appendix A.)

Pursuant to a mandate in the 1961 Single Convention, the INCB collects information and issues recommendations to national governments and international agencies. The Board collects estimates on the amount of controlled drugs needed for medical and scientific purposes, and on drug control measures generally, through an annual questionnaire. INCB members also conduct visits to some 20 countries annually, and the Board issues recommendations and observations to the governments of countries visited.[5] The INCB conducts trainings for drug control administrators, in collaboration with the UN Office on Drugs and Crime (UNODC) and the World Health Organization (WHO), and is authorized to recommend technical or financial assistance where appropriate.

If the INCB has objective reasons to believe that the UN drug conventions are being endangered, article 14 of the 1961 Single Convention permits the Board to recommend consultations with government officials, to propose that governments carry out their own national studies, or to publish an INCB report to draw attention to the country in question. The Board can also notify other countries of a failure in the drug control system or alert the UN Economic and Social Council (ECOSOC), a prominent UN body, when governments fail to explain or are otherwise deemed non-compliant with the conventions or with INCB recommendations. ECOSOC may further draw the attention of the UN General Assembly to such cases. (For greater detail on INCB powers under the UN drug conventions, see Appendix B.)

Beyond its formal remedial powers, the INCB engages in correspondence with national or local governments on issues of concern, and can request that governments account for their actions at INCB meetings in Vienna.

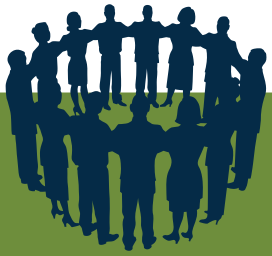

[INCB] meetings are closed to the public, and no minutes are made available.

These are held in three separate sessions, usually in February, May, and November, and total approximately five and a half weeks annually. The meetings are closed to the public, and no minutes are made available.

The most public and widely distributed account of INCB activities is its annual report, issued every year before the annual session of the Commission on Narcotic Drugs (CND) in Vienna (usually in March). According to the INCB, the annual report "provides a comprehensive survey of the drug control situation in various parts of the world" and includes estimated amounts of controlled drugs required by each country for medical and scientific uses.[6] These estimates are based on responses to the INCB's annual questionnaire. The annual reports also include a chapter on a current international drug policy issue, and sections on national policy developments in which the Board either praises or criticizes countries for their compliance with or deviance from the UN drug conventions.

Although the INCB has yet to recommend that ECOSOC report a member state to the UN General Assembly for failing to comply with the drug conventions, the Board pursues a vigorous campaign to draw attention

to developments in drug control and to ensure INCB prominence in international debates. "[I]ndicators of achievement" proposed by the UN for the 2004–2005 budgetary biennium, for example, included the expectation that the INCB would send more than 4300 letters to governments on matters related to the UN drug conventions, and would secure hundreds of media mentions of its annual report. Specifically, these indicators proposed:

- that the INCB would send 2600 letters to governments on matters related to the implementation of the 1961 and 1971 conventions, and 1700 letters related to the implementation of the 1988 Convention;
- that 170 governments would react to the Board's findings;
- that the international media would make 600 references to the INCB annual report; and
- that there would be 80 references by governments to the INCB annual report during the deliberations of the CND.[7]

In addition, the Board has a designated seat on the WHO Expert Committee on Drug Dependence, and the INCB president usually presents the Board's annual report to the CND (again, see Appendix A). INCB members speak at important UN meetings including General Assembly special sessions, regional meetings of WHO, and the World Health Assembly.

Expertise on HIV and drug policy

As the role of injection drug use in the spread of HIV has become clearer, INCB attention to drug use has also increased. The Board's annual report for 2005 notes the existence of or potential for drug-related HIV epidemics 18 times, referencing Afghanistan, Azerbaijan, China, Egypt, India, Indonesia, Kazakhstan, Lao People's Democratic Republic, Lesotho, Libya, Nepal, Nigeria, Malaysia, Myanmar, Russia, Swaziland, Thailand, Vietnam, Zambia, and Central Asia.[8]

The Board's expertise in HIV policy and programs, however, has not increased. The INCB includes experts in psychiatry, pharmacology, psycho-pharmacology, forensic medicine, and toxicology, but no AIDS experts. Of the extended biographies of INCB members included in the annual reports from 2001 through 2005, only one — that of Brian Watters, an advisor to the HIV/AIDS services of the Salvation Army in eastern Australia — includes any reference to experience with HIV policies or programs.[9] Furthermore, only one biography lists any publications on HIV/AIDS,[10] and a search of peer-reviewed medical, law and public-policy journals on HIV/AIDS shows no contributions by any member of the Board.

The INCB describes itself as a "quasi-judicial" body.[11] As such, the Board would be well served by members with expertise in international law. According to the biographies, only one of the current members is formally trained in the law, and his expertise is in drug law enforcement.[12] As INCB President A. Lourenço Martins noted to ECOSOC in 1999, "experience in applying and interpreting the law . . . is essential when analysing government performance under the treaties."[13] Martins' comment came as he reported to ECOSOC that as of 2000, the INCB would have no lawyers. Since then, the Board has included only one member with formal training and credentials in international law: Herbert Okun of the United States, who served from 1992 to 2001.

Despite its relative lack of expertise, the Board makes frequent pronouncements on international drug and health policies, often contradicting legal and health experts within and outside of the UN.

III. Statements and positions of the INCB

A. INCB and harm reduction

UN member states have twice unanimously endorsed their commitment to provide people at risk of HIV with comprehensive HIV/AIDS services, including harm reduction measures such as the provision of sterile syringes.[14] While no universal definition exists, "harm reduction" generally refers to efforts to reduce the adverse consequences of drug use among those who are unable or unwilling to abstain from illicit drugs.[15] In addition to the provision of sterile syringes, harm reduction measures include prescription of opiate substitutes to reduce illicit drug injection, information or peer counseling on safer injection and prevention of blood-borne illness, overdose prevention, and other measures to increase health and safety.

In the past decade, the explosion of HIV among injection drug users, and repeated studies showing the efficacy of measures such as syringe exchange and substitution treatment in decreasing HIV risk (and, in the case of substitution treatment, reducing demand for illicit opiates), have resulted in the formation of harm reduction networks in Latin America, the Caribbean, central and eastern Europe, and Asia. Harm reduction is a part of national strategies in countries as varied as Canada, Vietnam, Iran and Brazil.[16]

Emphasizing fear, downplaying science

The UN processes that produced consensus in favor of harm reduction were driven by a growing awareness that exploding HIV epidemics among people who inject drugs required new measures to contain infection. Yet the INCB, rather than acknowledging the urgent need for harm reduction to counter accelerating HIV epidemics, has repeatedly emphasized what it sees as the negative potential of the approach. Since 1993, the Board has issued repeated and unsubstantiated warnings that harm reduction has "diverted the attention (and in some cases, funds) of Governments from important demand reduction activities such as primary prevention or abstinence-oriented treatment."[17] Even when acknowledging that harm reduction can "play a part in a comprehensive drug demand reduction strategy,"[18] the INCB has been quick to sound notes of alarm. The foreword to the INCB annual report for 2003 lays out the INCB view:

> [Harm reduction] cannot . . . replace demand reduction programmes or be carried out at their expense. Most importantly, "harm reduction" can never be an end in itself, nor should it be the overall guiding principle behind national drug demand reduction policy. . . . While, in principle, measures to reduce harm in drug-dependent persons should not be seen as being in contradiction with the international drug control treaties, some so-called "harm reduction" approaches are not what they seem to be in that they cause more harm than they purport to reduce.[19]

Like many of the Board's statements on the subject of harm reduction, these assertions include a number of omissions. The statements make no reference to the harm caused by HIV. The INCB places scare quotes around "harm reduction," even though the term has been endorsed by UN member states. The Board cites no scientific evidence or specific examples to justify its assertion that some so-called harm reduction approaches cause more harm than good. The implication that harm reduction seeks to become the "overall guiding principle" behind national drug demand reduction is similarly unsubstantiated.

The INCB places scare quotes around "harm reduction," even though the term has been endorsed by UN member states.

The INCB's annual report for 2003 emphasizes other anxieties common to its publications, noting that harm reduction must never be pursued "at the expense" of "abstinence-oriented treatment" and that harm reduction measures "should not promote and/or facilitate drug abuse."[20] Again, no citations are offered to justify these concerns. The report fails to cite any of the multiple studies that show that harm reduction measures have not resulted in increased illicit drug use.[21] Having raised the specter of problems supposedly caused by harm reduction, the INCB ends by urging

governments to do the same:

> The Board calls on Governments that intend to include 'harm reduction' measures in their demand reduction strategies to carefully analyze the overall impact of such measures, which may sometimes be positive for an individual or for a local community while having far-reaching negative consequences at the national and international levels.[22]

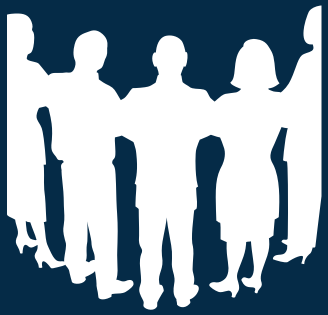

UN legal experts produced an internal document detailing multiple arguments that justified needle exchange, opiate substitution treatment, and safer injection facilities . . .

Ignoring expert legal analysis

In 2002, the Board asked the Legal Affairs section of the UN International Drug Control Programme (UNDCP) to review whether harm reduction interventions were compatible with the UN drug conventions. In September of that year, UN legal experts produced an internal document detailing multiple arguments that justified needle exchange, opiate substitution treatment, and safer injection facilities (SIF) under the terms of the conventions. The authors noted that all of these measures sought to reduce health and social problems, and alleviate human suffering as mandated by the conventions. Further, the report noted, although some argued that SIF were incompatible with convention obligations to prevent drug abuse, the same provisions obliged signatories to treat, rehabilitate and reintegrate those addicted to drugs. As the authors wrote:

> It would be difficult to assert that, in establishing drug-injection rooms, it is the intent of Parties [states] to actually *incite* or *induce* the illicit use of drugs, or even more so, to associate with, aid, abet or facilitate the *possession* of drugs. On the contrary, it seems clear that in such cases the intention of governments is to provide healthier conditions for IV drug abusers, thereby reducing their risk of injection with grave transmittable diseases and, at least in some cases, reaching out to them with counselling and other therapeutic options. Albeit how insufficient this may look from a demand reduction point of view, it would still fall far from the intent of committing an offence as foreseen in the 1988 Convention.[23]

No subsequent INCB annual report has referred to the legal findings of the UN experts. Three months after the INCB received the experts' report, a UN publication included an interview with INCB President Philip Emafo in which he noted that safer injection rooms and distribution of sterile injection equipment "amount to inciting people to use drugs" and contravened the conventions (for more on needle exchange and safer injection facilities, see below).[24]

Promoting opposition to harm reduction in the media

The INCB has not been completely immune to scientific or legal consensus. The INCB annual report for 2003 — perhaps as a corrective to earlier misstatements — stated clearly that "governments need to adopt measures that may decrease the sharing of hypodermic needles among injecting drug abusers in order to limit the spread of HIV/AIDS," and that the implementation of substitution and maintenance treatments "does not constitute any breach of the conventions."[25] However, a press release that accompanied the report qualified these supportive statements: "INCB Cautions on 'Harm Reduction' Measures in Drug Control," read the headline.[26] As late as 2005, U.S. Congressional opponents of needle exchange would claim, erroneously, that the INCB condemned the practice.[27]

B. INCB and opiate substitution treatment

Underemphasizing opiate substitution treatment

Opiate substitution treatment (OST), also known as opiate or opioid replacement therapy, provides patients with prescription medications such as methadone or buprenorphine to reduce cravings for heroin or other illicit opiates. It is among the best-researched and most effective forms of chemical dependence treatment. Delivered in health care facilities or physicians' offices, OST has been shown in repeated studies to reduce the risk of HIV, hepatitis C, overdose, and other complications of injection.[28] Significantly, as WHO has noted, OST is also an effective means of reducing demand for heroin,[29] and for increasing adherence to HIV medications.[30] The WHO Model List of Essential Medicines includes methadone and buprenorphine.[31]

The INCB annual report for 2003 clearly stated that OST did not breach the UN drug conventions and also noted that the INCB, over the years, had "discussed and confirmed quantities [of opiates] Governments have needed for such purpose."[32] In publicly available INCB reports and speeches, however, adequate discussion of the need for opiates for substitution treatment is rare. The INCB has failed to remark on the fact that estimates of need for methadone by countries such as Russia, Kazakhstan, Ukraine, and Vietnam have remained unchanged despite injection-driven HIV epidemics and skyrocketing rates of illicit opiate use.[33] Use of opiates to relieve pain from cancer and other chronic conditions is mentioned frequently in INCB speeches and reports.[34] OST, by contrast, is mentioned most often in INCB reports in the context of concern about diversion of methadone and buprenorphine to illicit markets.[35]

INCB documents and speeches by Board members routinely fail to note the ways that HIV epidemics heighten the importance of substitution treatment. In a speech to the World Health Assembly in May 2006, INCB President Philip Emafo did acknowledge the connection between OST and HIV prevention, though the comment was notable mainly for its tentativeness. Emafo said the INCB looked forward to working with WHO on the development of guidelines for the use of medications to manage opiate dependence, "which *might be* a component of community-based approaches for the prevention of HIV infection among injecting drug abusers" [emphasis added].[36] The INCB's annual report for 2005 noted that China had responded to HIV prevalence by implementing methadone programs. The observation, itself unusual in INCB reports, was made without expression of appreciation or praise.[37]

INCB documents and speeches by Board members routinely fail to note the ways that HIV epidemics heighten the importance of substitution treatment.

The INCB has demonstrated leadership and commitment in urging countries to expand medical opiate use for pain relief. The Board has worked with WHO and academic centers such as the University of Wisconsin's Pain and Policy Studies Group to highlight shortages in and facilitate greater access to opiates for pain relief. "The Board believes that the medical need for opiates is far from being fully satisfied in both less developed and developed countries," noted a 1996 INCB special report on medical uses of opiates.[38] The report, however, noted use for OST only in two instances. The first was the inclusion of addiction treatment in a list of medical applications for opiates that included treatment of diarrhea and cough, and use for veterinary purposes. The second was a table noting (without comment) that 45 percent of countries that provided information to the INCB allowed use of opiates for addiction treatment.[39] The report did not analyze either the lack of availability of methadone or buprenorphine to injection drug users, or the potential role of these medications in increasing social integration or reducing HIV risk. HIV/AIDS was mentioned only in the context of opiate use as palliative care for people living with AIDS. The annex listing non-governmental organizations consulted in the preparation of the report included no mention of HIV or harm reduction organizations.[40]

Failing to remark on country advances and failures

The INCB's inattention to substitution treatment is most noticeable — and most alarming — in visits to and

reports on countries where the treatment is illegal or unavailable. In Russia, for example, where the HIV epidemic is one of the fastest growing in the world and where UNODC estimates that 1.9 million people inject drugs, OST remains illegal.[41] Despite the INCB's mandate to ensure availability of opiates for medical uses, INCB representatives visited Russia in 2005 and made no public comment on the OST ban. Rather, the INCB annual report "noted the commitment of the government of the Russian Federation to addressing the problems of drug abuse and trafficking," expressed concern about "the large extent of drug abuse in the Russian Federation," and recommended measures to ensure sufficient funding for "prevention of drug abuse."[42] The report urged Russian authorities to promote the rational use of "opioids for the treatment of pain."[43]

Ironically, the INCB report also "encourages the Government to ensure adequate coordination and cooperation between the services providing treatment for drug addicts and HIV/AIDS services."[44] OST has served precisely this function in other countries, improving adherence to antiretroviral treatment for HIV and providing a means for ongoing, or directly observed, treatment for HIV and increased clinical benefit.[45] Thus, the INCB failed to address the very factor — illegality of opiate substitutes — that posed one of the largest obstacles to implementing its own recommendation.

The INCB's inattention to substitution treatment is most noticeable — and most alarming — in visits to and reports on countries where the treatment is illegal or unavailable.

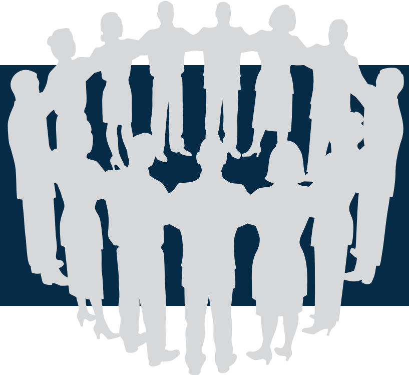

The INCB has failed to remark on lack of methadone or buprenorphine in numerous countries where HIV prevention and care is severely hampered by lack of OST. The INCB annual report for 2005 noted the link between heroin injection and HIV in Kazakhstan, but said nothing about that country's failure to honor its pledge to provide OST using its grant from the Global Fund to fight AIDS, Tuberculosis, and Malaria.[46] In Ukraine, which has the highest national HIV prevalence in Europe and an HIV epidemic concentrated among injection drug users, the Board noted that methadone remained prohibited in 2005, and then unhelpfully observed that countries were entitled to observe stricter restrictions on methadone than those required by the UN drug conventions.[47] In its comments on India, the Board recognized the growing problem of heroin injection, but did not comment on the lack of methadone.[48] Afghanistan, a country that receives extensive attention from the Board as the world's primary producer of opium, has no OST, a situation lamented by some experts but not commented upon by the INCB.[49]

Use of heroin is growing in sub-Saharan Africa,[50] but OST is largely unavailable across the continent.[51] The Board made visits in 2005 to Lesotho and Swaziland, where more than 20 percent of adults are HIV-positive, and noted that it "is concerned that the current situation could be further exacerbated by insufficient drug abuse prevention programmes."[52] The INCB made no mention of insufficient addiction treatment programs. INCB President Philip Emafo also spoke in August 2006 to the WHO Regional Committee for Africa — an exceptional chance to reach African ministers of health — yet did not mention HIV or OST.[53]

The INCB has also missed opportunities to praise advances in provision of OST. China, for example, has won international praise for its 2004 decision to expand methadone availability.[54] Malaysia, facing an HIV epidemic heavily concentrated among drug users, has begun to provide OST through private and government clinics.[55] Ukraine began to offer buprenorphine in 2005 to reduce HIV infections and increase adherence to HIV medications. While INCB annual reports sometimes note the implementation of new OST programs, the Board has yet to comment favorably on the public health benefits of increased availability of medications proven to reduce HIV risk and demand for illicit drugs.

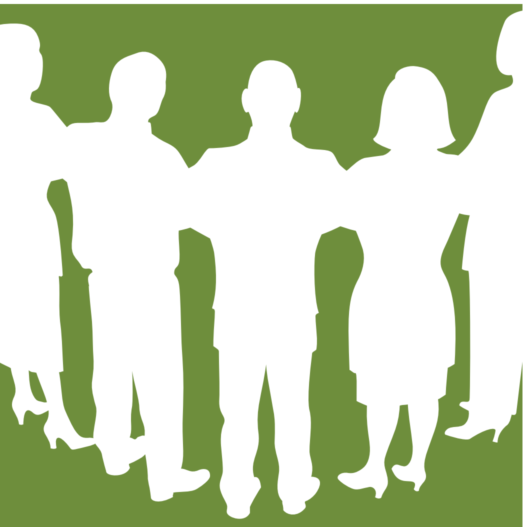

[T]he INCB — despite the absence of legal criteria for its stance — has used its influence to pressure countries to reverse or delay implementation of safe injection facilities . . .

Stressing drug control at the expense of public health

The INCB has urged governments to increase controls on OST in ways likely to undermine national commitment to expansion of the treatment. Observing that worldwide consumption of methadone has increased by almost three and a half times in the last decade, the INCB's annual report for 2005 raises concern about potential diversion of methadone and urges governments to consider restricting access through supervised methadone consumption, short dispensing intervals, and central registration of all opioids prescribed for medical use.[56] This recommendation ignores the multiple research articles and government guidelines that have noted the potential positive impact of allowing take-home doses of methadone as a means of retaining some patients in treatment.[57]

The Board also considers buprenorphine almost exclusively in terms of its potential for diversion, repeatedly referencing its use for illicit purposes. The INCB annual report for 2005 urges WHO to consider tightening the control status of buprenorphine to reduce diversion, but makes no reference to the substantial impact rescheduling would have on access to substance abuse treatment or HIV prevention. The Board notes concerns about buprenorphine diversion in France,[58] while failing to mention that measures such as widespread buprenorphine prescription and needle exchange programs have reduced HIV infections among people who inject drugs to nearly zero.[59]

C. INCB and safer injection facilities

Safer injection facilities (SIF), sometimes called supervised injection facilities or supervised injection rooms, are centers where people may inject drugs using clean equipment under the supervision of medically trained personnel. At the close of 2005, SIF operated in Switzerland, Germany, the Netherlands, Australia, Spain, Norway, Canada and Luxembourg, with regulations in place for them in Portugal. SIF have been the object of extensive research and rigorous evaluations. Benefits of SIF documented in peer-reviewed research include fewer deaths from overdose, more referrals to addiction treatment and related services, and a reduction in syringe sharing (and therefore in the risk of HIV and hepatitis transmission). Studies have also shown no increase in drug-using behavior among those using SIF and, in the case of the Canadian facility, no disruption of public order.[60]

Condemnation without legal justification

Despite this evidence, the INCB has issued multiple statements decrying what it called "drug injection rooms" as "a step in the direction of drug legalization".[61] For example, its report for 2002, declared that:

> The Board believes that any national, state or local authority that permits the establishment and operation of drug injection rooms or any outlet to facilitate the abuse of drugs (by injection or any other route of administration) also facilitates illicit drug trafficking. . . . By permitting drug injection rooms, a Government could be considered to be in contravention of the international drug control treaties by facilitating, aiding and/or abetting the commission of crimes involving illegal drug possession and use, as well as other criminal offences, including drug trafficking.[62]

The INCB has reiterated this position repeatedly as countries have opened SIF,[63] at times offering additional, unsubstantiated critiques. In his presentation to the CND in 2000, the INCB president characterized SIF as "shooting galleries" and places where "drug abusers are allowed to abuse illicit drugs obtained from the illicit market under supervision and under, *supposedly*, hygienic conditions" [emphasis added].[64] The implication

that SIF are unhygienic is not documented in any publicly available account. The INCB's allegation that SIF facilitate or promote drug use is similarly unsupported by the multiple studies that have examined it.[65]

As noted earlier (see *INCB and harm reduction* above), legal experts from the UNDCP have differed with the INCB's assertion that SIF are in violation of the UN drug conventions. The attorneys general or chief prosecutors of Germany, the Netherlands, Switzerland, and Slovenia, among other countries, have considered the question of SIF in relation to the drug conventions; all have come to conclusions similar to that of the UNDCP's legal experts.[66] Nonetheless, the INCB has remained steadfast in its insistence on the illegality of SIF, without any legal justification for its view.

Silencing other UN actors

Equally alarmingly, the INCB — despite the absence of legal criteria for its stance — has used its influence to pressure countries to reverse or delay implementation of safe injection facilities, and even to threaten UN personnel who support such facilities. For example, the Board's annual report for 2000 singled out Australia as a country where some jurisdictions "unfortunately" chose to support "heroin injection rooms" that violate treaty obligations.[67] The annual report for 2001 reiterated this censure, even as it highlighted the problem of heroin overdose in Australia,[68] a problem that has been reduced by medically supervised injection.[69] Referring to New South Wales, where a safer injection site had been established, the Board urged Australia and other governments to "ensure that all of its states comply fully with the provisions of the international drug control treaties."[70] Similarly, Canada was singled out by the Board in its annual report for 2003, which suggested that opening North America's first SIF in Vancouver violated UN drug conventions.[71]

In May 2006, Stephen Lewis, then the UN Secretary-General's Special Envoy for HIV/AIDS in Africa, spoke favorably about Vancouver's safer injection facility at the International Conference on the Reduction of Drug Related Harm. Lewis had visited the facility, known as Insite, shortly before the conference. Noting research that documented Insite's success in helping people who use drugs reduce HIV-related risk, he encouraged the Canadian government to renew the facility's exemption from federal drug laws, and encouraged other cities in Canada to consider SIF.[72] The speech was widely reported by the press.

Lewis received a telephone call the next day from Koli Kouame, the secretary of the INCB. According to Lewis, Kouame told him that his ill-informed support of "opium dens" would be reported to the UN Secretary-General (at whose pleasure Lewis served as Special Envoy) and that the INCB would take up the matter of Lewis' alleged indiscretion.[73] In a letter to UN Secretary-General Kofi Annan dated May 18, 2006, INCB President Philip Emafo expressed shock at Lewis' remarks, noting: "The Board did not believe that any officer of the United Nation [*sic*] could have made such statements." Emafo demanded that Lewis retract his statements, noting that the UN drug conventions were established "precisely to eliminate places, such as opium dens, where drugs could be abused with impunity." Emafo reiterated the Board's view that SIF violated the "most fundamental principle" underlying all of the UN drug conventions: that drug production and consumption be "limited exclusively to medical and scientific purposes".[74] Emafo did not mention findings by UN legal advisors and other experts to the contrary.

D. INCB and sterile syringe programs

Sterile syringe programs, including syringe exchange, are a central element of HIV prevention in epidemics linked to drug injection. WHO has reviewed the vast literature on sterile syringe programs that finds them effective in reducing HIV and hepatitis C transmission without promoting or encouraging drug use.[75] The unanimous declarations that resulted from the UN General Assembly Special Session on HIV/AIDS in 2001 and the UN General Assembly High-Level Meeting on AIDS in 2006 included commitments from all UN member states to ensure access to sterile injecting equipment.[76] A 2000 UN "system-wide" position paper asserted the support of all UN agencies to measures ensuring access to sterile injecting equipment as part of a comprehensive HIV prevention strategy.[77]

Misrepresenting law and science

As noted earlier, INCB President Philip Emafo said in a 2002 interview — after legal experts had decided that needle exchange was not a violation of the UN drug conventions — that "to promote drug use illicitly through the giving out of needles . . . would, to me, amount to inciting people to abuse drugs, which would be contrary to the provisions of the conventions."[78] This continued a tradition of misstatements by INCB presidents on the subject of syringe exchange. In a 1999 speech to ECOSOC, then-President A. Lourenço Martins asserted that the Board was concerned that the distribution of syringes in prison was a measure "not in strict accordance with the provisions of the conventions."[79] Nine countries provide sterile syringes in prison, and at least four more have plans to do so. Evaluations of many of these programs have concluded that they effectively prevent HIV and hepatitis C, increase the safety of prison staff, and do not promote initiation of drug injection.[80] No published legal analysis of prison-based sterile syringe programs suggests these programs violate the UN drug conventions. Indeed, recent UNODC guidelines on prisons and HIV/AIDS, launched in 2006 at the XVI International AIDS Conference in Toronto, endorse sterile syringe programs in prisons where such programs are available outside penal institutions.[81]

As noted earlier, the Board's annual report for 2003 did issue a corrective, noting that governments "need to adopt measures that may decrease the sharing of hypodermic needles . . . in order to limit the spread of HIV/AIDS."[82] Having conceded the usefulness of sterile injection equipment in one sentence, however, the Board casts doubt on the approach in the next, stressing that "prophylactic measures should not promote and/or facilitate drug abuse."[83] The insinuation that providing sterile injection equipment might promote drug use was made without reference to abundant scientific studies that find this not to be the case.[84]

E. INCB and the human rights of people who use drugs

Many UN documents, notably the General Assembly resolutions on HIV/AIDS, the *UN International Guidelines on HIV/AIDS and Human Rights*, and the 2005 UNAIDS position paper on strengthening global HIV prevention, have emphasized the importance of programs that build on and reinforce human rights protections — including for people who use drugs — as a condition for effectively addressing HIV.[85] The 25-year response to HIV has shown clearly that policies and programs that respect the human rights of marginalized populations are more likely to reach those they seek to help, since they are unlikely to provoke fear and marginalization.

The INCB has been dismissive of this central tenet, reinforcing a disregard for the human rights of people who use drugs through errors of omission and commission. The Board's annual reports refer regularly to "drug abusers": since the UN drug conventions prohibit all but medical use, the report for 2001 noted, any illicit use is abuse.[86] INCB analysis has focused on a human right defined by the Board but absent from all international covenants, the "human right to be protected from drug abuse."[87] INCB member Herbert Okun, speaking as the "INCB rapporteur" to a 1998 gathering convened by the organization European Cities Against Drugs, emphasized that "drug abusers" are frequently violent because of the effects of their addiction, and that "people who do not take drugs have a right to be protected from this kind of behaviour."[88] Okun noted further that "[d]rug addicts are unable to make free decisions for their future."

Silence on abuses at the national level

The Board's analysis in the annual report for 2003 included a recommendation for a balanced approach to law enforcement. Rather than "heavy-handed police crackdowns on drug abusers and communities where illicit drug markets thrive," the report called for "sensitive law enforcement, through community policing initiatives."[89] At the national level, however, the INCB itself has demonstrated little sensitivity to overly harsh policing, and has failed to recognize that illicit drug use should not be synonymous with forfeiture of due process or human rights protections. An example is the INCB visit to Thailand in 2004, made some months after a widely criticized drug war resulted in the arrest or internment of more than 50 000 citizens — including many with no history of drug use — and the killing of more than 2500 people in what human rights groups said were professionally executed assassinations.[90] Human Rights Watch, Amnesty International, academic

experts and Thai civil society groups protested the “war on drugs,” noting that small-scale drug users were being caught in the crossfire and that HIV prevention services for drug users were being severely compromised.[91]

Reporting on this 2004 visit (meant to examine the impact of the Thai government’s campaign) the INCB expressed appreciation of a government effort to investigate the killings — an effort that many human rights experts had deemed completely inadequate.[92] The Board said it trusted that the Thai government would continue to provide information regarding the “progress of those investigations.”[93] Though it noted with appreciation that some government officials had been prosecuted for corruption, the INCB report did not mention that no government officials had been investigated for the killings. The Board expressed no concern about the thousands arrested in the name of drug treatment or the impact of the government campaigns on health programs. The Board’s report also noted that the government actions had resulted in decreased methamphetamine use,[94] a statement that ignored the human cost of the means used to achieve this end. In the face of an abusive drug crackdown that undermined humane drug treatment and HIV prevention, the Board could not muster a statement about protection of the human rights of drug users.

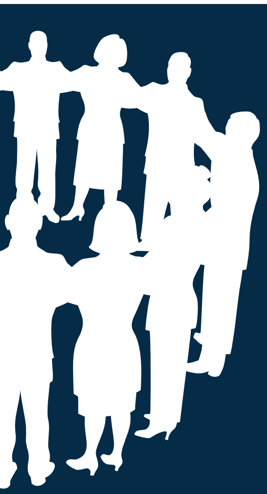

[The INCB] has failed to recognize that illicit drug use should not be synonymous with forfeiture of due process or human rights protections.

In 2004, Bulgaria passed one of the world’s harshest drug laws, criminalizing possession of drugs in even the tiniest amount, with mandatory incarceration for as long as 10 to 15 years. A 2005 study published by the Brussels-based Center for the Study of Democracy found that both drug injection (measured in number of injections per person per day) and use of non-sterile syringes increased markedly after the law came into force.[95] Fear of police caused people to inject more frequently in remote or abandoned neighborhoods, where they were unlikely to have access to sterile syringe programs or medical assistance in case of overdose. These results echo the conclusions of studies from other countries on the impact of harsh policing and repressive laws.[96] The European Commission criticized Bulgaria for the harshness of its law and raised questions about the law’s provision for incarceration for minor crimes.[97]

In contrast, the INCB’s report of its 2005 visit to Bulgaria noted that the country showed “political commitment and the will to deal with drug abuse,” and that the “national drug control legislation and the institutional framework to carry out these policies and implement that legislation are well developed.”[98] The Board did not express concern that the harshness of the law might discourage people who use drugs from seeking chemical dependence treatment and other health services, such as HIV prevention or treatment.

Similarly, long-term, mandatory incarceration for possession of even the smallest amounts of drugs in Russia has been linked to widespread human rights violations, including police brutality and violations of due-process rights. Wholesale incarceration has also been identified as a factor in helping the Russian HIV epidemic become the fastest growing in the world.[99] In 2004, Russia reformed its laws to reduce indiscriminate incarceration, a change that the head of the UNODC recognized as a positive step.[100] Unfortunately, less than a year later, the Russian government backtracked on this reform and reinstituted harsh penalties, including mandatory incarceration for possession of small amounts of drugs.[101] The INCB visited Russia in May 2005, while the reversal was being finalized. The Board’s account of the visit makes no reference to this issue or to the impact of the policy on human rights or HIV. Rather, the INCB praised “the commitment of the Government of the Russian Federation to addressing the problems of drug abuse and trafficking.”[102]

Since 1990, China has made June 26, the UN’s International Day Against Drug Abuse and Illicit Trafficking, the culmination of an annual week of police and judiciary action in which hundreds are sentenced to death and

scores are executed for crimes of drug smuggling, trafficking, and production. Sentences have been announced at show trials attended by crowds of thousands chanting "kill, kill," and in some years executions — which numbered 222 in 1996 alone — have been broadcast on television or conducted in public.[103] While the INCB annual report for 2003 noted that many countries had repealed the death penalty, the Board did not comment on these show trials and executions, conducted on a UN day of awareness. The Board did observe that the practice of execution could complicate international proceedings such as extraditions, particularly when the requesting state permits capital punishment and the requested state does not.[104]

HIV prevention and other basic health services for people who use drugs are seriously impeded in other ways by police action. In Russia, Kazakhstan, Ukraine, Thailand, and the United States, for example, human rights researchers have documented the targeting of people who use drugs for arrest or harassment at needle exchange sites, or when they seek to purchase syringes legally in pharmacies.[105] In these countries, as well as others such as Bangladesh, police have threatened or harassed needle exchange workers.[106] Laws that criminalize possession of sterile syringes have undermined the utilization and effectiveness of HIV prevention efforts in many countries, including parts of the United States.[107] Though the INCB's reports could urge protection of staff and clients of needle exchange programs from police harassment, they have not done so.

Dissonance with the UN

The INCB's portrayal of "drug abusers" unable to participate meaningfully in decision-making underscores the Board's distance from the commitment to the human rights of people who use drugs and others vulnerable to HIV, which is emphasized in numerous UN documents. For example, the policy paper on intensifying HIV prevention approved by the UNAIDS governing board in June 2005 noted that HIV prevention and treatment for people who use drugs should include peer outreach — that is, people who use drugs reaching out to other people who use drugs — and that HIV/AIDS programs "must be based on promoting, protecting and respecting the human rights of drug users."[108] This observation echoed in more specific terms the unanimous declarations from the UN General Assembly in 2001 and 2006 for participation of those living with and vulnerable to HIV/AIDS in programs and policy decision-making.[109] UNAIDS Asia Regional Director J.V.R. Prasada Rao, in a major address in 2006, recognized that respect by HIV organizations for the human rights of people who use drugs required real participation and partnership with drug users: "that doesn't just mean polite meetings and shared recommendations; it means a real flow of resources to drug user networks . . ."[110]

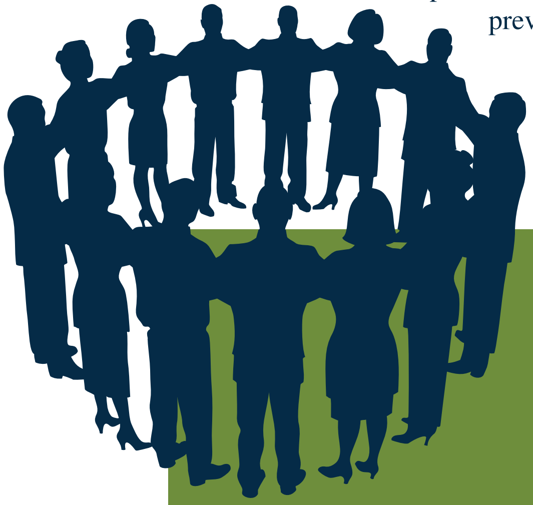

Though the INCB's reports could urge protection of staff and clients of needle exchange programs from police harassment, they have not done so.

The INCB, while independent, should still be accountable. Statements that suggest that people who use drugs cannot be regarded as responsible enough to protect their health and the health of others reinforce the same stigma that the UN system has pledged to combat.

IV. Impartiality, independence and accountability at the INCB

The tension between independence and lack of accountability is evident in many issues related to the INCB. The scope and high profile of INCB activities makes it clear that the Board plays an important role in shaping the international response to drugs and HIV. The INCB Secretariat, which sits behind locked doors in the building that houses the UNODC, emphasizes the need for independence, impartiality, and protection against outside influence. The bulk of INCB operating expenses, including the salaries of the 28 Secretariat staff and the approximately US$700,000 the INCB spends every two years for travel expenses, are provided from the UN core resources drawn from the pooled dues of UN member states.[111] This helps ensure that the Board is not subject to undue pressure from any country or group of states.

INCB members themselves receive only token compensation of US$1 per year, and are elected by secret ballot by ECOSOC. Under the provisions of the 1961 Single Convention, 10 of the 13 INCB members are nominated by UN member states from persons with "knowledge of the drug situation in the producing, manufacturing and consuming countries" and three positions on the Board are reserved for experts with "medical, pharmacological or pharmaceutical experience" nominated by WHO. The 1961 Single Convention also enjoins ECOSOC to ensure geographic diversity on the Board and to ensure that INCB members are "persons who, by their competence, impartiality and disinterestedness, will command general confidence."[112] INCB members serve five-year terms and may be re-elected. In 1998, INCB President Hamid Ghodse told ECOSOC, responsible under the UN drug conventions for acting upon violations of impartiality or independence by the INCB, that the Board had "instituted an internal procedure whereby the impartiality and disinterestedness of its members are guaranteed throughout their stay in the Board."[113]

Country influence

A review of actions by Board members suggests the need to strengthen both the independence and accountability of the INCB. From 1995 to 2005, 11 of the 34 persons who served on the Board had been delegates from their countries to the CND, including four who headed their country's delegation.[114]

Russia and the case of methadone

While INCB members ostensibly represent their own interests, rather than those of their member states, the influence of their country's policies is often reflected in their statements. A clear example of this difficulty is found in the pronouncements on methadone of INCB member and former Russian health minister Tatyana Dmitrieva. As noted earlier, Russia remains one of the few countries in the world to ban substitution treatment. In 2005, Dmitrieva was one of five public figures in Russia to sign a memorandum entitled "No to methadone programs in Russia" (*Nyet metadonovym programmam v Rossii*), published in the *Meditsinskaya Gazeta*, a widely read newspaper for medical professionals. Dmitrieva was identified in the memorandum as an INCB member. The text contained numerous inaccuracies and half-truths about methadone.[115] The memorandum insinuates incorrectly that WHO has opposed methadone therapy for most of its history and that the CND has rejected methadone as a tool for treating heroin addiction. It also recounts deaths from methadone overdose in Lithuania that experts in that country could not corroborate, and cites research by authors whose names could not be found in any medical database. The authors included many assertions of the ineffectiveness or dangers of methadone, without citing evidence to support their claims.

Scientists from the United States, the United Kingdom, Iran, the Czech Republic, Italy, Australia, Albania, Switzerland, Croatia, Germany, Canada and France issued a heavily referenced, point-by-point reply to this statement, correcting the article's many errors.[116] The INCB, despite the use of its name in the memorandum, has not published a correction.

Influence of the INCB at the UN

Though the INCB does not feel bound to document the sources for its conclusions, the Board is deeply committed to urging UN bodies to adopt them. For example, the INCB makes regular presentations to ECOSOC, the main coordinating body in the UN for economic and social policy and questions of economic development. The INCB normally releases its annual report shortly before the annual meeting of the CND and presents its findings at the CND meeting. As noted above, multiple references to INCB annual reports by national delegations at the CND are regarded as a performance indicator for the INCB Secretariat.[117] Countries singled out for censure in INCB reports frequently issue rebuttals from the floor of the CND. Of 104 resolutions passed by the CND between 1996 and 2005, 21 (or about 20 percent) made reference to the INCB. The UNODC's annual *World Drug Report* regularly acknowledges the INCB and makes reference to INCB data and observations.

The INCB also interacts with other UN bodies. Since 1969, a member of the INCB has attended every meeting of the WHO Expert Committee on Drug Dependence (ECDD), the UN body that is charged with determining the degree of "abuse liability" of drugs and to recommend appropriate control measures to the CND.[118] The INCB's emphasis on diversion and abuse of opiate substitutes, and its failure to consider questions of effective chemical dependence treatment, find expression in ECDD debates. In 2003, for example, INCB concern about buprenorphine diversion helped provoke a discussion about whether controls on the medication should be increased by rescheduling it under the 1961 Single Convention (rather than the 1971 Convention). In 2006, the INCB and several concerned member states again raised the question of rescheduling buprenorphine. The ECDD decided against it, noting that treatment with buprenorphine "usefully replaces injections and . . . limits transmission of HIV/AIDS and other blood-borne infections among injecting drug users by reducing use of contaminated needles."[119]

Lack of public guidelines on attribution of statements or corrections of fact

Misrepresentations of fact on the permissibility of needle exchange under the UN drug conventions by INCB presidents Emafo and Martins, and on methadone by INCB member Tatyana Dmitrieva, highlight a larger issue: the difficulty of determining whether Board members are speaking for themselves or for the Board, and the lack of publicly available mechanisms through which the INCB can be called upon to correct errors of fact. No publicly available documents explain how questions of scientific expertise are judged by the Board, or whether there are mechanisms to correct the record if Board members make statements unsupported by scientific or legal evidence.

A culture of secrecy

In proposing reform and increased transparency of the UN in 2002, then-Secretary-General Kofi Annan made improving UN engagement with civil society a central theme.[120] By 2006, this effort had reached the UN Security Council, whose president proposed opening deliberations to representatives of the UN, member states, and civil society organizations through measures including informal briefings, more publication of information on websites, open meetings, and invitations from member states to NGOs to meetings where their presence would enhance deliberations.[121]

While the INCB is an independent body, it is — as is evident throughout these pages — integrally related to UN drug control efforts, and staffed and paid for by the UN. Most of the INCB's deliberations are no more sensitive than those of the Security Council.

Without compromising its independence, the INCB might enhance its credibility and accountability by instituting formal and informal measures of the kind now pledged by the Security Council. Specifically, the Board should allow UN agencies and civil society groups a greater role in providing information for and monitoring the results of INCB activities. Although WHO and UNODC staff do attend sessions of INCB meetings, for the most part they do so only as observers. The INCB has no formal or public mechanism to include the input of civil society groups, whose participation has proven essential in formulating effective

responses to HIV and drug use. The INCB does not publicize its country visits in advance, offer criteria for how countries are selected for visits, or hold public forums while on these visits. Countries visited by the INCB undoubtedly value the chance to discuss their drug policies in confidence. But the secrecy surrounding the planning of country visits and the lack of any mechanism for input from health professionals or non-governmental experts impede their completeness and effectiveness. The INCB does not publicize the many letters it writes to countries about its concerns, presumably to ensure constructive dialogue. The secrecy, however, also insulates the Board from healthy dialogue about its focus and priorities.

Existing UN mechanisms could easily be used to improve INCB engagement with civil society representatives. In Vienna, for example, the INCB could easily call upon the NGO forum that is held during each session of the CND to organize NGO participation in some of its sessions.

V. Conclusions and recommendations

In various arenas, the INCB has shown leadership and vision that extends beyond drug control to another central tenet of the UN drug conventions: the alleviation of human suffering. INCB efforts to ensure adequate supplies of controlled medications to relieve the pain of cancer and HIV patients, for example, demonstrate the importance of Board leadership, the value of collaboration with UN agencies, and the ways in which commitment at the Secretariat in Vienna can help enable dialogue at the national level. In discussions of programs to provide alternative livelihoods for farmers growing illicit crops, too, the Board has urged such measures as "full participation of the crop growers, their families and the community" in the design of solutions, and the importance of recognizing the ways in which "economic vulnerability, hopelessness and disempowerment may put them at higher risk of drug abuse and addiction."[122]

The INCB is clearly aware that the changing understanding of the impacts of drug use requires changes in emphasis. The Board's questionnaire to measure progress since the 1998 UN General Assembly Special Session on Drugs, for example, refers to "addicts" rather than abusers, includes attention to the question of chemical dependence treatment and availability of rehabilitation facilities, and seeks to ascertain whether countries have measures in place to reduce drug-related harm.[123] On various occasions, the Board has acknowledged the complexity of drug addiction and its link to social, cultural and economic factors, and proposed methods of engaging community members and diminishing punishments to enable reintegration of people who use drugs into society.[124]

The Board has not, however, recognized that what is true for people who grow illicit crops is also true for people who use drugs: they are entitled to meaningful participation in programs and policy-making that affect them, and such participation would improve the effectiveness of efforts to help alleviate problems related to illicit drugs. This failure is particularly problematic with respect to HIV/AIDS, where the importance of participation by affected communities and attention to human rights has been repeatedly emphasized by UN agencies and national governments.

Unfortunately, as these pages document, the INCB remains closed to reason on many issues of critical importance to the prevention and treatment of HIV.

. . . the INCB remains closed to reason on many issues of critical importance to the prevention and treatment of HIV.

Recommendations

The following actions are needed if the INCB is to work in step with other global actors to forge a meaningful and effective response to HIV/AIDS.

Greater attention to availability and quality of treatment for chemical dependence: The INCB should regularly assess and report on the availability and quality of treatment for chemical dependence, in keeping with international best practice and the UN drug conventions. In countries where HIV is significantly linked to the injection of opiates, such assessment should include the degree of coordination of HIV/AIDS and drug treatment programs, the availability of opiate substitution treatment, and estimates of the need for such treatment. Where OST is illegal or inaccessible, the INCB should work with both health and drug authorities to overcome barriers to its effective use. The Board should emphasize the compatibility of OST with the provisions of the UN drug conventions in countries such as Russia, where national authorities and INCB members have suggested that the illegality of methadone is consistent with the conventions. The Board should recommend technical or financial assistance to help countries ensure that they provide legal opiates in quantities needed for all medical uses, including addiction treatment. Performance indicators for the INCB should include mechanisms for the monitoring and availability of chemical dependence treatment, such as the number of letters sent to governments that focus on the availability of methadone and other opiate substitutes as part of national HIV/AIDS programs, and the number of media mentions that highlight the INCB's concern for human rights violations perpetrated in the name of drug treatment and rehabilitation.

Greater documentation of legal conclusions and assertions about the effectiveness of harm reduction strategies: The absence of references to published literature in INCB annual reports and statements undermines the INCB's credibility. The INCB should document its findings in the way that WHO technical reports are documented. Observations that come from country visits should be noted as such, and comments on public health interventions that have been the subject of peer-reviewed scientific research should acknowledge that research. INCB reports should review and incorporate the findings of the 2002 memorandum of the UNDCP's Legal Affairs Section on the compatibility of safer injection facilities and sterile syringe programs with the international conventions. The Board should also review and reference other published legal analyses and research documenting the effectiveness of SIF and sterile syringe programs in providing referrals to addiction treatment and reducing HIV risk without increasing illicit drug use.

Greater transparency and accountability, particularly through engagement with UN member states and civil society: The annual report of the INCB sets the tone for discussion in the CND and is widely covered in the media. UN member states and civil society should have regular opportunities to exchange ideas with the Board and to offer responses to the findings in these reports. Governing bodies of UN agencies and of large institutions such as the Global Fund to fight AIDS, Tuberculosis and Malaria have instituted formal mechanisms for civil society representation in their deliberations. The INCB should do the same, if only for its own information. The INCB should give advance notice of country visits, and allow time to exchange views with local policy-makers and civil society organizations representing the interests of people who use drugs and others affected by HIV/AIDS. It would be helpful for the Board to issue a public justification for the closed nature of its meetings, deliberations and correspondence. This analysis should include an assessment of the possibility of conducting informal open sessions with UN, national governments and non-governmental bodies.

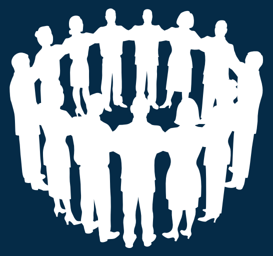

The UN Secretary-General should commission an independent evaluation of the INCB's work.

Greater efforts to ensure HIV expertise on the Board: Given the relevance of Board actions on the global HIV epidemic, its membership should include people with expertise in HIV. The World Health Organization should ensure that its five nominees for Board membership include at least three people who have significant and demonstrable expertise in drug policy and HIV/AIDS. ECOSOC should request UN member states to consider HIV/AIDS expertise in putting forward their nominations for the Board. ECOSOC should also encourage UN member states to include persons with expertise in international law among their nominees.

Guidelines for independence of Board members: The Board should articulate, and ECOSOC should evaluate, public guidelines to make clear when Board members are speaking for the INCB or as individuals, and how misstatements of fact can be corrected.

Independent evaluation of the INCB: The UN Secretary-General should commission an independent evaluation of the INCB's work. This evaluation should include consultation with all UN agencies that are co-sponsors of UNAIDS, and experts and organizations working on HIV prevention among people who use drugs. The evaluation should consider the impact of INCB opinions and reprimands on national policy and the impact of INCB's work on HIV policy. It should also investigate the independence and impartiality of INCB members and the criteria used by UN member states and WHO to nominate Board members. The oversight responsibilities outlined in the 1961 Single Convention on Narcotic Drugs make this an appropriate task for the Secretary-General.

Appendix A: INCB and the mechanisms of global drug control

There are three key global entities with worldwide drug control responsibilities:

UN Commission on Narcotic Drugs (CND)

Established in 1946, this rotating group of 53 UN member states elected by the UN Economic and Social Council (ECOSOC) is the principal policy-making body on drug control in the UN system. The CND's annual session is open to attendance by other countries, UN agencies and non-governmental organizations that have ECOSOC consultative status.

UN International Drug Control Programme (UNDCP)

Now a part of the **UN Office on Drugs and Crime (UNODC)**, this agency is "mandated to assist UN member states in their struggle against illicit drugs, crime and terrorism." Its core activities include "normative work to assist States in the ratification and implementation of the international treaties, the development of domestic legislation on drugs, crime and terrorism, and the provision of secretariat and substantive services to the treaty-based and governing bodies."[125] UNODC is a co-sponsor of UNAIDS.

International Narcotics Control Board (INCB)

The INCB is "the independent and quasi-judicial control organ for the implementation of the United Nations drug conventions," with the "responsibility to promote government compliance with the provisions of the drug control treaties."[126] The INCB is a body of 13 people who serve in their personal capacities. The INCB Secretariat is provided by the UN, its expenses are covered by the UN, and it holds meetings in UN facilities — but it is a body ostensibly independent of the United Nations system and of any government.

UN drug conventions

The current global system for drug control rests upon three international conventions:

- 1961 Single Convention on Narcotic Drugs (as amended by a 1972 protocol)
- 1971 Convention on Psychotropic Substances
- 1988 Convention Against Illicit Traffic in Narcotic Drugs

The ***1961 Single Convention*** consolidated and replaced a series of previous treaties. It aims to control the cultivation, production, manufacture, export, import, distribution of, trade in, use and possession of opiates (such as morphine, heroin and methadone), cannabis and cocaine, limiting these activities "exclusively to medical and scientific purposes" (article 4c). The controlled substances are listed in a series of schedules that classify medications according to medical benefit, abuse potential and level of restriction.

The 1961 Single Convention also requires states that have ratified it to adopt measures to ensure that the above-mentioned activities are "punishable offences when committed intentionally" and that "serious offences shall be liable to adequate punishment particularly by imprisonment or other . . . deprivation of liberty" but "subject to the constitutional limitations" of the state (article 36:1a). Where "abusers of drugs" — "abusers" is the common reference to people who use drugs in the conventions — have committed such offences, the state "may provide, either as an *alternative to conviction or punishment* or in addition to conviction or punishment, that such abusers shall undergo measures of treatment, after-care, rehabilitation and social reintegration" (article 36:1b). It also enjoins governments to "give special attention to and take *all practicable measures* for the prevention of abuse of drugs and for the early identification, treatment, education, after-care, rehabilitation and social reintegration of the persons involved" (article 31:1, emphasis added).

The ***1971 Convention*** follows the same basic approach as the 1961 Single Convention, but with a focus on substances not covered by the earlier treaty, particularly synthetic preparations of stimulants, depressants and hallucinogens, again set out in a series of schedules. As with the 1961 Single Convention, it makes provision for the use of these substances for medical and scientific purposes, subject to controls to prevent diversion into illicit channels.

The ***1988 Convention*** went further toward the goal of global drug prohibition. It requires that state parties "legislate as necessary to establish a modern code of criminal offences relating to the various aspects of illicit trafficking and to ensure that such illicit activities are dealt with as serious offences by each State's judiciary and prosecutorial authorities." While the primary focus of the 1988 Convention is on *trafficking*, it also includes a requirement that states, subject to their own legal systems, establish measures to criminalize "the possession, purchase or cultivation of narcotic drugs or psychotropic substances *for personal consumption*" (emphasis added, article 3.2), but not beyond the level of criminalization already required in the previous conventions. The 1988 Convention also states clearly that while governments are required to criminalize the possession of illicit drugs for personal consumption, they have latitude when it comes to the appropriate penalty.[127]

None of the conventions contains any language specifically on reducing the harms associated with drug use, including preventing infectious disease. The first two conventions were conceived before HIV and the hepatitis C virus were known, and the 1988 Convention was developed before there was global awareness of the importance of injection drug use as a determinant of HIV.[128] Thus, the conventions do not address directly such harm reduction programs as sterile syringe programs and methadone therapy that seek to mitigate the health and social consequences of drug use without insisting on abstinence from drugs.

Appendix B: The role of the INCB as specified in the UN drug conventions

Articles cited here are from the 1961 Single Convention (as amended in 1972) unless otherwise noted.

The INCB "in cooperation with governments and subject to the terms of this Convention, shall endeavour to limit the cultivation, production, manufacture and use of drugs to an adequate amount required for medical and scientific purposes, to ensure their availability for such purposes and to prevent illicit cultivation, production and manufacture of, and illicit trafficking in and use of, drugs" (article 9.4).

The INCB will gather and determine the manner of gathering information from countries on estimated quantities of controlled drugs needed for medical and scientific purposes (articles 12 and 19). The Board is also allowed to make such estimates where countries do not provide data on them (article 12.3) or amend estimates provided if it has good evidence supporting such amendment (article 12.5). Similar powers are delegated to the INCB with respect to statistics on the production and manufacture of controlled drugs and seizures and disposal of drugs (articles 13 and 20).

If the INCB, on the basis of all evidence it collects, "has objective reasons to believe that the aims of this [1961] Convention are being seriously endangered," it may request explanations of a government or "propose to the Government concerned the opening of consultations" on the matter. If the problem persists, the INCB may call upon the government to adopt "remedial measures". It may further propose to the government that a study be carried out "in its territory by such means as the government deems appropriate." If none of these measures is effective, the INCB may bring the matter to the attention of ECOSOC, the CND, and the governments that are party to the Convention (article 14). ECOSOC may further draw the attention of the UN General Assembly to such cases where governments do not seem to be cooperating. The INCB may also make recommendations to UN agencies that a given state requires technical assistance (article 14 bis). The 1971 Convention extended similar duties to the INCB with respect to synthetic psychotropic substances (article 19) and the 1988 Convention did the same with respect to its trafficking provisions (article 22).

The Board is mandated to produce an annual report on its work to be submitted to ECOSOC through the CND (article 15, 1961 Conv.).

If the Board finds that manufacture or importation of a controlled substance in a country exceeds a reasonable quantity for scientific and medical purposes (where calculation of a reasonable quantity is made by criteria specified in article 21 of the 1961 Single Convention), it may notify other countries that may be affected by this excess (article 21).

While the INCB does not then have the authority to impose sanctions or to adjudicate the conventions in any binding way, it is authorized to make recommendations about the implementation of the UN drug conventions, call on governments to do better and notify UN bodies and other countries about the nature of its findings. Its annual report often includes criticism of countries for their role in what the Board perceives to be inadequate enforcement of or attention to the spirit of the conventions. In addition, the Board issues letters of concern to governments, which are not made public.

To fulfill its duties, the INCB for its part notes that it undertakes the following activities:[129]

> Administers a voluntary system of government reports on narcotic drugs, psychotropic substances and licit activities involving drugs to help governments achieve, among other things, "a balance between supply and demand."
>
> Monitors and promotes measures taken by governments to prevent diversion of substances used in the illicit manufacture of controlled drugs so as to determine whether the scope of the 1988 Convention needs to be modified.

Analyzes information provided by governments, UN agencies and other international organizations to ensure that the provisions of the drug treaties are adequately carried out and recommends remedial measures.

Maintains a “permanent dialogue” with governments to assist their compliance with their treaty obligations and recommend, “where appropriate, technical or financial assistance to be provided.”

In collaboration with UNDCP and WHO, conducts training programs for drug control administrators, particularly in developing countries.

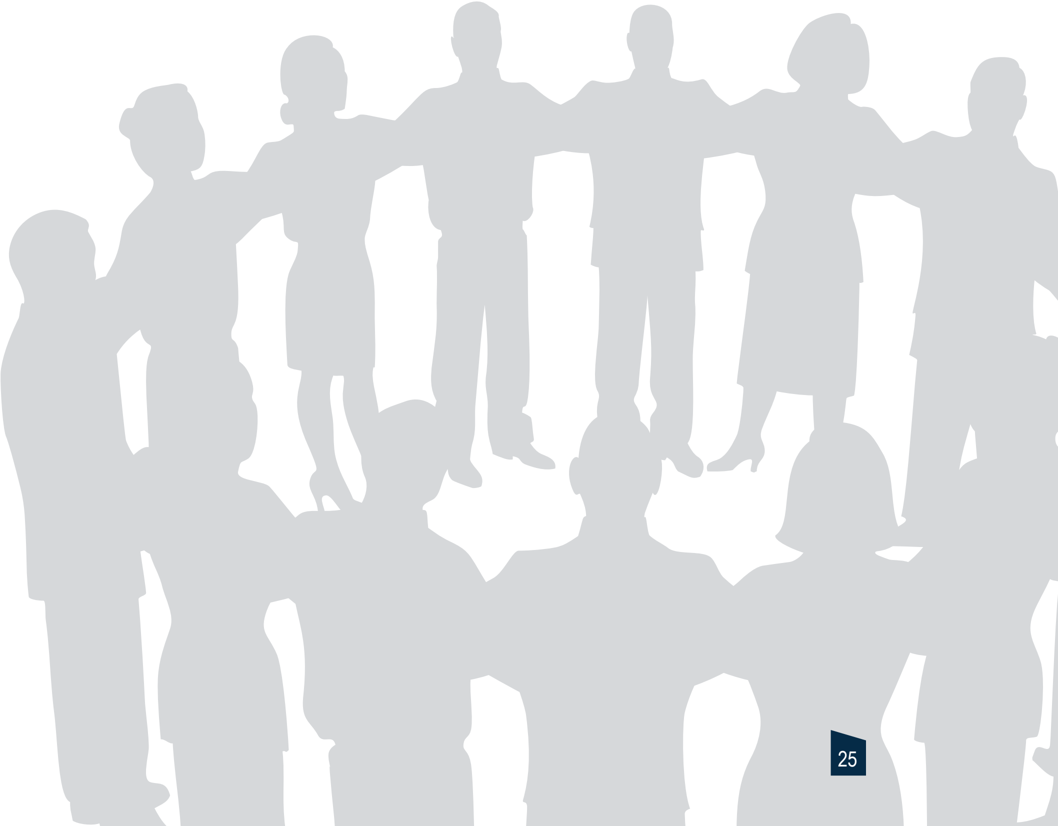

References

[1] See, for example, Agence France Presse, February 27, 2002, and BBC coverage of INCB reports (visit http://search.bbc.co.uk and search for "INCB").

[2] UNAIDS, *2006 Report on the Global AIDS Epidemic* (Geneva: United Nations Joint Programme on HIV/AIDS, 2006).

[3] See, for example, ongoing activities of the UNODC. Available at http://www.unodc.org/unodc/drug_demand_hiv_aids_activities.html. See also UN General Assembly, *Proposed programme budget for the biennium 2006–2007*, Section 16—International drug control, crime prevention and criminal justice, A/60/6(Sect.16), 2.

[4] UN General Assembly, ibid., para 16, 19.

[5] International Narcotics Control Board, *Report of the International Narcotics Control Board for 2005*, E/INCB/2005/1 (Vienna, 2006), para 185. Available at www.incb.org.

[6] International Narcotics Control Board, "Mandates and Functions" section of the INCB website. Available at www.incb.org.

[7] UN General Assembly, *Proposed programme budget for the biennium 2004–2005*, section 17 — International drug control (A/58/6), 14–17.

[8] D Wolfe and J Cohen. *Selective concern: The 2005 report of the International Narcotics Control Board and the need for accountability.* New York, Open Society Institute, 2006.

[9] International Narcotics Control Board, *Annual report for 2005*, Annex II, 104–112.

[10] Ibid., 104. Dr. Joseph Asare, a psychiatrist from Ghana, was an author of an article reporting results of a survey on HIV and drug use in Ghana.

[11] Available at www.incb.org. Consulted December 15, 2006.

[12] International Narcotics Control Board, *Annual report for 2005*, 105. This member is Madan Mohan Bhatnagar of India.

[13] A Lourenço Martins (INCB president). Speech to the substantive session of ECOSOC, Geneva, July 1999.

[14] UN General Assembly, *Declaration of commitment on HIV/AIDS* (A/RES/S-26/2), August 2, 2001; and UN General Assembly, *Political declaration on HIV/AIDS* (A/RES/60/262), June 15, 2006.

[15] See, for example, British Department for International Development, *Harm reduction: Tackling drug use and HIV in the developing world* (Department for International Development, 2005). Available at http://www.dfid.gov.uk/pubs/files/hivharmreduction2005.pdf; International Harm Reduction Development Program, *Saving lives by reducing harm: HIV prevention and treatment for injecting drug users* (New York: Open Society Institute, 2006). Available at http://www.soros.org/initiatives/health/focus/ihrd/articles_publications/publications/saving_20060818.

[16] See, e.g., Government of Brazil, Conselho Nacional Antidrogas, *Politica nacional sobre drogas*. Section on harm reduction (Redução dos danos sociais e à saúde). Available at www.senad.gov.br; Government of Canada — Health Canada, *Canada's drug strategy*. Available at http://www.hc-sc.gc.ca/ahc-asc/activit/strateg/drugs-drogues/index_e.html; Socialist Republic of Vietnam, 11th National Assembly, Session no. 9. *Law on prevention and control of HIV/AIDS* (article 21, "HIV/AIDS harm reduction interventions"), 2006. English translation of the law obtained by and on file with the Canadian HIV/AIDS Legal Network.

[17] International Narcotics Control Board, *Report of the International Narcotics Control Board for 2000*, E/INCB/2000/1 (Vienna, 2001), para 446.

[18] Ibid.

[19] International Narcotics Control Board, *Report of the International Narcotics Control Board for 2003*, E/INCB/2003/1 (Vienna, 2004); International Narcotics Control Board, *Background release*, February 27, 2004; International Narcotics Control Board, *Annual report for 2005*, para 185. Available at www.incb.org.

[20] International Narcotics Control Board, *Annual report for 2003*, paras 219 and 221.

[21] See, for example, World Health Organization, *Effectiveness of sterile needle and syringe programming in reducing HIV/AIDS among injecting drug users — Evidence for Action Technical Paper* (Geneva, 2004); U.S. National Academies of Science, Institute of Medicine, *Preventing HIV infection among injecting drug users in high risk countries* (Washington, D.C. 2006).

[22] International Narcotics Control Board, *Annual report for 2003*, paras 27–28.

[23] UNDCP Legal Affairs Section, *Flexibility of treaty provisions as regards harm reduction approaches*, E/INCB/2002/W.13/SS.5 (UNDCP Legal Affairs Section, September 2002), paras 27–28 (emphasis in original).

[24] Interview with Philip O. Emafo, MD. *UNODC Update*, December 2002. Available at www.unodc.org/unodc/newsletter_2002-12-31_1_page004.html.

[25] International Narcotics Control Board, *Annual report for 2003*, paras 221–222.

[26] International Narcotics Control Board, press release no. 4, March 3, 2004 (Vienna: United Nations Information Service).

[27] Reps. Tom Davis and Mark Souder, U.S. House of Representatives. Letter to Andrew Natsios, administrator of the U.S. Agency for International Development, February 11, 2005. See U.S. House of Representatives, Committee on Government Reform, Subcommittee on Criminal Justice, Drug Policy and Human Resources. *Harm reduction or harm maintenance: Is there such a thing as safe drug abuse?* Serial no. 109–36 (U.S. Government Printing Office, February 16, 2005), 43–45. In this letter, Reps. Davis and Souder assert that the INCB "sharply criticized 'harm reduction' measures such as needle exchange programs" in its annual report for 2003. In the same hearings in the US Congress, Peter Bensinger, a former administrator of the US Drug Enforcement Administration, said he had worked with the INCB for many years and noted, "I would doubt if the [INCB] would like to sanction needle exchange rooms any more than they sanctioned opium dens…". See page 56 of *Harm reduction or harm maintenance: Is there such a thing as safe drug abuse?*

[28] See, for example, DJ Bellis. "Reduction of AIDS risk among 41 heroin addicted female street prostitutes: effects of free methadone maintenance." *Journal of Addictive Diseases* 1993; 12: 7–23; B Meandzija et al. HIV infection and cocaine use in methadone maintained and untreated intravenous drug users. *Drug and Alcohol Dependence* 1994; 36: 109–13; D Metzger et al. "Human immunodeficiency virus seroconversion among intravenous drug users in and out of treatment: an 18 month prospective follow-up." *Journal of Acquired Immune Deficiency Syndrome* 1993; 6: 1049–56. See also WHO/UNODC/UNAIDS, *Substitution maintenance therapy in the management of opioid dependence and HIV/AIDS prevention: position paper* (Geneva: United Nations, 2004).

[29] WHO/UNODC/UNAIDS, *Substitution maintenance therapy*, 13.

[30] Ibid., 24.

[31] World Health Organization, *WHO model list of essential medicines* (revised March 2005). Available at http://mednet3.who.int/EMLib/index.aspx .

[32] International Narcotics Control Board, *Annual report for 2003*, para 222.

[33] International Narcotics Control Board, *Estimated world requirements of narcotic drugs for 2005* (Vienna, 2006); International Narcotics Control Board, *Estimated world requirements of narcotic drugs for 2001* (Vienna, 2002). Both available at www.incb.org.

[34] See, for example, International Narcotics Control Board, *Availability of opiates for medical needs* (special report prepared pursuant to Economic and Social Council resolutions 1990/31 and 1991/43) (Vienna, 1996), iii, 5–6 ff.; International Narcotics Control Board, *Annual report for 2005*, para 88; International Narcotics Control Board, *Report of the International Narcotics Control Board for 2004*, E/INCB/2004/1 (Vienna, 2005), para 137; International Narcotics Control Board, *Annual report for 2003*, 173–174; statements by INCB presidents to the 42nd session of CND in 1999, the 43rd session in 2000, the 1999, 2000, 2001 and 2004 substantive sessions of ECOSOC, the 59th session of the World Health Assembly in 2006, the 46th Directing Council of the Pan American Health Organization in 2005, and the WHO Regional Committee for Africa in 2006. All speeches available at www.incb.org.

[35] See, for example, International Narcotics Control Board, *Annual report for 2004*, para 93; International Narcotics Control Board, *Annual report for 2005*, paras 116, 138, 495, 562, 652.

[36] PO Emafo (president, INCB). Statement to the 59th session of the World Health Assembly, May 2006, Geneva. Available at www.incb.org/incb/speeches/.

[37] International Narcotics Control Board, *Annual report for 2005*, para 467.

[38] International Narcotics Control Board, *Availability of opiates for medical needs*, iii.

[39] Ibid., iii and 6. When the medical uses of opiates were noted in a 2000 speech to ECOSOC by then-INCB president A. Lourenço Martins, he mentioned anesthetic, analgesic, veterinary and dental purposes with no mention of treatment of narcotics addiction. See A. Lourenço Martins (president, INCB). Statement to the 43rd session of the Commission on Narcotic Drugs. Vienna, March 2000.

[40] International Narcotics Control Board, *Availability of opiates for medical needs*, Annex II, 23.

[41] For HIV in Russia, see UNAIDS, *2006 report on the global AIDS epidemic*. Chapter on eastern Europe and central Asia is available at http://data.unaids.org/pub/EpiReport/2006/06-Eastern_Europe_and_Central_Asia_2006_EpiUpdate_eng.pdf.

[42] International Narcotics Control Board, *Annual report for 2005*, para 587.

[43] Ibid., para 619.

[44] Ibid., paras 572 and 618.

[45] See, for example, JP Moatti et al. "Adherence to HAART in French HIV-infected injecting drug users: the contribution of buprenorphine drug maintenance treatment. The Manif 2000 study group." *AIDS 2000*; 14(2): 151-5.

[46] International Narcotics Control Board, *Annual report for 2005*, paras 517, 559, 560; see also IRIN News, Kazakhstan: fight against HIV/AIDS continues (Nairobi: UN Office for the Coordination of Humanitarian Affairs) August 23, 2005. Available at www.irinnews.org/print.asp?ReportID=48722.

[47] International Narcotics Control Board, *Annual report for 2005*, para 584.

[48] Ibid., para 503. In November 2006, the UNAIDS representative in India called for the government to register methadone for OST. See *Associated Press*, As India tops with world's largest number of HIV cases, new strategy targets drug users, November 29, 2006.

[49] See, for example, CS Todd et al. Correction: Drug use and harm reduction in Afghanistan. *Harm Reduction Journal* 2005; 2:20.

[50] UNODC, *World drug report 2006* (Vienna, 2006), esp. 67, 75.

[51] According to INCB data, of the 51countries in North Africa and sub-Saharan Africa that provided estimates of need for narcotic drugs, 13 estimated some need for methadone. Of these, all but three said their estimated need was under 40 grams. See International Narcotics Control Board, *Estimated world requirements of narcotic drugs for 2005*.

[52] International Narcotics Control Board, *Annual report for 2005*, para 300.

[53] PO Emafo (president, INCB). Statement to the WHO Regional Committee for Africa, Addis Ababa, August 28, 2006. Available at http://www.incb.org/incb/speeches/speech_who_2006-08-28.html.

[54] J Cohen. "HIV/AIDS in China: changing course to break the HIV-heroin connection." *Science* 2004; 304(5676):1434–35.

[55] M Mazlan et al. "New challenges and opportunities in managing substance abuse in Malaysia." *Drug and Alcohol Review* 2006; 25(5): 473–8.

[56] International Narcotics Control Board, *Annual report for 2005*, para 75. See also Wolfe and Cohen, *Selective concern*.

[57] See, e.g., Health Canada, *Best practices: methadone maintenance treatment* (Ottawa: Health Canada, 2002), 44 and 58: "Programs should balance the advantages of ensuring compliance and having regular contact with clients/patients with the need for flexible, client/patient-centred treatment that takes into account the realities of clients'/patients' lives." Research has shown that flexible take-home doses are an important factor in patient retention. Also, regulations should specify how these requirements are to be judged. Examples of tools for assessment are listed in New Zealand Ministry of Health, *Opioid substitution treatment: New Zealand practice guidelines*, February 2003, 28. See also "Carry Policy" guidelines in The College of Physicians of Ontario [Canada], *Methadone maintenance guidelines* (Toronto, 2001), 18–21.

[58] International Narcotics Control Board, *Annual report for 2005*, para 138.

[59] EuroHIV, *HIV/AIDS surveillance in Europe — end-year report 2005* (Saint-Maurice: Institut de veille sanitaire, 2006), esp. 58.

[60] See, for example, Medically Supervised Injecting Centre Evaluation Committee, *Final report on the evaluation of the Sydney Medically Supervised Injecting Centre* (Sydney, Australia: MSIC Evaluation Committee, 2003); T Kerr et al. "Safer injection facility use and syringe sharing in injection drug users." *Lancet* 2005; 366(9482): 316–318; E Wood et al. "Attendance at supervised injecting facilities and use of detoxification services." *New England Journal of Medicine* 2006; 354(23):2512–2514; J Kimber et al. "Drug consumption facilities: an update since 2000." *Drug and Alcohol Review* 2003; 22:227–233; E Wood et al. "Changes in public order after the opening of a medically supervised safer injecting facility for illicit injection drug users." *Canadian Medical Association Journal*, 2004; 171: 731–734; and European Monitoring Centre for Drugs and Drug Addiction, *European report on drug consumption rooms* (Lisbon, February 2004).

[61] International Narcotics Control Board, "Drug injection rooms — Not in line with international conventions," press release no. 5, February 23, 2000 (Vienna: United Nations Information Service). Available at www.incb.org.

[62] International Narcotics Control Board, *Annual report for 2000*, paras 176–177.

[63] International Narcotics Control Board, *Annual report for 2005*, para 590 scolds Norway, which opened a supervised injection facility in Oslo in 2005.

[64] A Lourenço Martins (president of INCB). Statement to the 43rd session of the Commission on Narcotic Drugs, March 2000.

[65] See, for example, E Wood et al. "Impact of a medically supervised safer injecting facility on drug dealing and other drug-related crime." *Substance Abuse Treatment, Prevention and Policy* 2006; 1:13–17; T Kerr et al. "Impact of a medically supervised safer injection facility on community drug use patterns: a before and after study." *British Medical Journal* 2006; 332:220-222.

[66] See, e.g., citations from decisions in Germany, Slovenia, Switzerland and the Netherlands in Canadian HIV/AIDS Legal Network. *Legislating for health and human rights: Model law on drug use and HIV/AIDS* (module 4: Supervised drug consumption facilities), p 13, note 29. 2006. Available at www.aidslaw.ca/modellaw.

[67] International Narcotics Control Board, *Annual report for 2000*, para 525.

[68] International Narcotics Control Board, *Report of the International Narcotics Control Board for 2001*, E/INCB/2001/1 (Vienna, 2002), paras 559, 565.

[69] See, for example, MW Tyndall et al. "Attendance, drug use patterns and referrals made from North America's first supervised injection facility." *Drug and Alcohol Dependence* 2006; 83(3): 193–198.

[70] International Narcotics Control Board, *Annual report for 2001*, para 559.

[71] International Narcotics Control Board, *Annual report for 2003*, paras 325–326.

[72] The federal Minister of Health of Canada on September 1 issued an announcement that Insite would continue to operate on a trial basis until December 2007 and that during the extended trial period more research on its impact would be conducted, and no new applications for SIF would be considered. See Health Canada, News release: "No new injection sites for addicts until questions answered says Minister Clement," September 1, 2006.

[73] Stephen Lewis, personal communication.

[74] P Emafo (president, INCB). Letter to Kofi Annan (on the subject of Stephen Lewis' statements), 18 May 2006.

[75] World Health Organization, *Effectiveness of sterile needle and syringe programming in reducing HIV/AIDS among injecting drug users — Evidence for Action Technical Paper* (Geneva, 2004).

[76] UN General Assembly, *Declaration of commitment on HIV/AIDS*, August 2, 2001, para 52; and UN General Assembly, *Political declaration on HIV/AIDS*, June 15, 2006, para 22.

[77] United Nations High-Level Committee on Programme, *Preventing the transmission of HIV among drug abusers: A position paper of the United Nations System*, E/CN/7/2002/CRP.5 (Vienna, 2001). Available at http://www.cicad.oas.org/en/Resources/UNHIVaids.pdf.

[78] Interview with Philip O. Emafo MD. *UNODC Update*, December 2002.

[79] A Lourenço Martins. Statement to the Substantive Session of the Economic and Social Council, New York, 1999.

[80] R Lines et al. *Prison needle exchange: Lessons from a comprehensive review of international evidence and experience*, 2nd ed. (Toronto: Canadian HIV/AIDS Legal Network, 2006), esp. i–iii. Available at www.aidslaw.ca/prisons.

[81] UNODC with WHO and UNAIDS. *HIV/AIDS prevention, care, treatment and support in prison settings: A framework for an effective national response* (Vienna, 2006), 24.

[82] International Narcotics Control Board, *Annual report for 2003*, para 221.

[83] Ibid.

[84] WHO, *Effectiveness of sterile needle programmes*, summarizes the extensive evidence on this question.

[85] UNAIDS, *Intensifying HIV prevention: UNAIDS policy position paper* (Geneva, 2005). This paper issued by the member states of UNAIDS' governing body calls for HIV prevention approaches "based on promoting, protecting and respecting the human rights of drug users" (p 34).

[86] International Narcotics Control Board, *Annual report for 2001*, foreword by INCB President H. Ghodse, iii.

[87] H Ghodse (president of the INCB). Speech to the UN General Assembly Special Session on the World Drug Problem, New York, June 1998.

[88] HS Okun. International drug control policy on the eve of the 21st century: Statement by Ambassador HS Okun. Stockholm, May 1998.

[89] International Narcotics Control Board, *Annual report for 2003*, para 41.

[90] Human Rights Watch, *Not enough graves: The war on drugs, HIV/AIDS and violations of human rights — Thailand* (New York, 2004).

[91] See, for example, Ibid.; Amnesty International, *Thailand's anti-drug policy should not be killing people*, March 2004, available at http://web.amnesty.org/pages/tha-010304-action-eng; and T Vongchak et al. The influence of Thailand's 2003 "war on drugs" policy on drug use among injection drug users in Chiang Mai, Thailand. *International Journal of Drug Policy* 2005; 16:115–121. Thailand has been praised for its national AIDS program, which has resulted in remarkable declines in HIV transmission among sex workers, but drug users have generally not been the focus of government HIV/AIDS efforts and continue to be heavily affected by HIV. See UNAIDS, *Country update — Thailand*, 2006. Available at http://www.unaids.org/en/Regions_Countries/Countries/thailand.asp.

[92] See, for example, letter from Asian Human Rights Commission Executive Director Basil Fernando to UN Secretary-General Kofi Annan (on the occasion of Annan's visit to Thailand), May 23, 2006. Available at www.ahrchk.net.

[93] International Narcotics Control Board, *Annual report for 2004*, para 388.

[94] Ibid., para 389.

[95] T Bezlov, *Heroin users in Bulgaria one year after outlawing the dose for "personal use": Law changes and new risks* (Brussels: Centre for the Study of Democracy, 2005).

[96] T Kerr et al. "The public health and social impacts of drug market enforcement : A review of the evidence." *International Journal of Drug Policy* 2005; 16: 210–220.

[97] Europe criticizes Bulgaria on drug laws. *Sofia Echo*, June 12, 2005.

[98] International Narcotics Control Board, *Annual report for 2005*, para 614.

[99] Human Rights Watch. *Lessons not learned: human rights abuses and HIV/AIDS in the Russian Federation* (New York, 2004); and United Nations Development Programme. *Reversing the epidemic: Facts and policy options — HIV/AIDS in Central and Eastern Europe and the CIS* (Bratislava: UNDP, 2004), esp. 49-61.

[100] AM Costa. Opening remarks to the Commission on Narcotic Drugs, Vienna, 7 March 2005. Available at http://www.unodc.org/unodc/speech_2005-03-07_1.html.

[101] L Levinson. "Russian Federation: Going backwards on drug policy." *HIV/AIDS Policy and Law Review* 2005; 10(2): 27–28.

[102] International Narcotics Control Board, *Annual report for 2005*, para 572.

[103] See, for example, Xinhua News Agency, "Executions after anti-drugs rallies in Yunnan," June 28, 1990; United Press International, "Seven drug traffickers executed in south China," June 28, 1991; Agence France Presse, "Some 50 drug traffickers executed in China," June 27, 1992; Agence France Presse, "More than 100 drug traffickers executed in Yunnan," June 28, 1993; *The Times* (London), "Drug example," June 27, 1994; Deutsche Presse-Agentur, "104 people executed in China," June 26, 1995; Agence France Presse, "222 people executed in one day in China's anti-drug crackdown," June 27, 1996; Deutsche Presse-Agentur, "China executes 28 for drug trafficking, violent crime," June 27, 1997; AAP Information Services, "China: Almost 1,900 executed in China last year." September 10, 1998; Agence France Presse, "China executes Pakistani national as crackdown on drug crime continues," June 28, 1999; Agence France Presse, "China executes total of 52 over drugs in a week," June 30, 2000. *The Guardian* (London), "Chinese executions mark UN's anti-drugs day: Crackdown on traffickers comes as number of HIV-Aids cases soars," June 27, 2001; Deutsche Presse-Agentur, "China executes at least 82 to mark anti-drugs day." June 26, 2002; Deutche Presse-Agentur, "China Executes 26 People for Drug Crimes," June 26, 2003; Agence France Presse, "China executes 17 drug traffickers," June 26, 2004 and "Eighteen executed in China," June 25, 2004; Xinhua News Agency, "Dozens of drug dealers given to death penalty in China's combat against drug-related crimes," June 25, 2004; Agence France Presse, "Asia marks anti-drug day with executions, destruction of narcotics," June 26, 2006; and Agence France Presse, "Twenty seven executed in China on anti-drug day." June 27. 2006.

[104] International Narcotics Control Board, *Annual report for 2003*, paras 213–214.

[105] See, for example, Human Rights Watch, *Fanning the flames — how human rights abuses are fueling the AIDS epidemic in Kazakhstan* (New York, 2003), 32–33; Human Rights Watch, *Rhetoric and risk: human rights abuses impeding Ukraine's fight against HIV/AIDS* (New York, 2006), 34–38; Human Rights Watch, *Injecting reason: Human rights and HIV prevention for injection drug users* (New York, 2003), 20–24; Human Rights Watch, *Lessons not learned* (Russia report), 33–35; Human Rights Watch. *Not enough graves* (Thailand report), 40–41.

[106] Ibid. See also Human Rights Watch, *Ravaging the vulnerable: Abuses against persons at high risk of HIV infection in Bangladesh* (New York, 2003), 45–46.

[107] Human Rights Watch, *Injecting reason*, 23–25.

[108] UNAIDS, *Intensifying HIV prevention: UNAIDS policy position paper* (Geneva, 2005), 34.

[109] UN General Assembly, *Declaration of commitment on HIV/AIDS*, August 2, 2001, paras 32, 33; UN General Assembly, *Political declaration on HIV/AIDS*, June 15, 2006, para 20.

[110] JVRP Rao, *From advocacy to implementation: challenges and responses to injecting drug use and HIV*. Plenary speech at XVII International Conference on the Reduction of Drug-Related Harm, Vancouver, April 30, 2006.

[111] UN Economic and Social Council, *Consolidated budget for the biennium 2006–2007 for the United Nations Office on Drugs and Crime*, E/CN.7/2005/12, September 27, 2005.

[112] United Nations. *Single convention on narcotic drugs, 1961* (as amended by the 1972 Protocol amending the Single Convention), article 9. Available at http://www.unodc.org/pdf/convention_1961_en.pdf.

[113] H Ghodse (president of the INCB). Statement to the substantive session of ECOSOC, New York, 1998.

[114] Persons who served as INCB members in 1995–2005 after having been delegates from their countries to the CND include Edouard Babayan (Russian Federation — also head of delegation), Dil Jan Khan (Pakistan, also delegation head), A Lourenço Martins (Portugal), Oskar Schroeder (Germany, delegation head), Elba Torres Graterol (Venezuela), Chinmay Chakrabarty (India), Alfonso Gomez Mendez (Colombia), Nüzhet Kandemir (Turkey), Madan Mohan Bhatnagar (India, head of delegation), Robert Lousberg (Netherlands), and Rainer Schmid (Austria). Philip Emafo (Nigeria) was chairman of the tenth Special Session of the CND in 1988.

[115] V Krasnov et al. *"Nyet metadonovym programmam v Rossii"* (No to methadone programs in Russia). *Meditsinskaya Gazeta* no. 29, 30 March 2005, 7. Slightly altered version available in Russian at http://www.healthinstitute.ru/catalog/memorandum.htm.

[116] C Aceijas et al. "Say no to methadone" memorandum: Correcting the record (memo and open letter). Available at http://www.soros.org/initiatives/health/focus/ihrd/news/methadone_2006/corrections_20060511.pdf#search=%22no%20to%20methadone%20memorandum%20correcting%22.

[117] UN General Assembly, *Proposed programme budget 2004–2005*, 15.

[118] Description of the Committee's mandate available on the web site of WHO at http://www.who.int/substance_abuse/right_committee/en/index.html. The Committee's published reports document the participation of an INCB representative since 1969.

[119] "Topics of current interest," *WHO Drug Information* 2006; 20(3): 190. Available at http://www.who.int/druginformation/.

[120] UN General Assembly, *Strengthening of the United Nations: an agenda for further change (report of the Secretary-General)*, A/57/387, September 9, 2002, 24.

[121] UN Security Council, *Note by the President of the Security Council (plus Annex)*, UN doc. 06-42943, 2006.

[122] International Narcotics Control Board, *Annual report for 2005*, para 23(g).

[123] International Narcotics Control Board, *Evaluation of implementation of the international drug control treaties (questionnaire)*, 2007. Available at http://www.incb.org/pdf/questionnaire/Questionnaire_Eng.doc.

[124] See, for example, International Narcotics Control Board, *Annual report for 2004*, para 2, and International Narcotics Control Board, *Annual Report for 2003*, para 59.

[125] See "About Us" on UNODC website at http://www.unodc.org/unodc/en/about.html.

[126] International Narcotics Control Board, "Role of INCB" on the Board's web site at www.incb.org.

[127] Commentary on the United Nations Convention Against Traffic in Narcotic Drugs and Psychotropic Substances 1988 (December 1988).

[128] International Harm Reduction Development Program, *Unintended consequences: Drug policies fuel the HIV epidemic in Russia and Ukraine* (New York: Open Society Institute, 2003), 5.

[129] International Narcotics Control Board, "Mandates and functions" section of INCB website. Available at http://www.incb.org/incb/en/mandate.html.

Canadian HIV/AIDS Legal Network | Réseau juridique canadien VIH/sida

Canadian HIV/AIDS Legal Network
1240 Bay Street, Suite 600
Toronto, Ontario, Canada M5R 2A7
Telephone: +1 416 595-1666 | Fax: +1 416 595-0094
E-mail: info@aidslaw.ca
Website: www.aidslaw.ca

OPEN SOCIETY INSTITUTE
Public Health Program

International Harm Reduction Development Program
Open Society Institute
400 West 59th Street
New York, NY 10019 United States of America
Telephone: +1 212 548-0677 | Fax: +1 212 428-4617
E-mail: ihrd@sorosny.org
Website: www.soros.org/harm-reduction
