# Supplementary material for: Closed to reason: time for accountability for the International Narcotic Control Board
Source: Harm Reduct J. 2007 May 8;4:13. doi: 10.1186/1477-7517-4-13 (PMC1871577; doi:10.1186/1477-7517-4-13)
Supplement: Additional file 2 — Closed to Reason: The International Narcotics Control Board and HIV/AIDS Key Findings. Russian translation of key findings of the above report. [file 1477-7517-4-13-S2.pdf]

Основные выводы и рекомендации

Роль наркополитики видоизменилась после того, как был сформирован Международный комитет по контролю за наркотиками (МККН), на 13 членов которого была возложена ответственность за выполнение конвенций ООН по контролю за наркотиками. Сейчас, когда за пределами Африки примерно в 30 процентах случаев инфекция передается через потребление инъекционных наркотиков, наркополитика является ключевым фактором, определяющим успех или провал международной реакции на распространение ВИЧ/СПИДа. Большая база данных научных исследований подтверждает, что для проведения профилактики ВИЧ-инфекции важны такие меры, как эффективное лечение химических зависимостей и предоставление стерильных шприцев. Перед МККН, который подчеркивает свою объективность, независимость и опору на фактические данные, открывается историческая возможность помочь остановить эпидемию ВИЧ среди потребителей инъекционных наркотиков, возникающую и распространяющуюся с огромной скоростью во многих странах мира.

Вместо этого Комитет сам стал препятствием на пути эффективных программ профилактики и лечения ВИЧ-инфекции и химических зависимостей. Годовые отчеты МККН изобилуют упущениями и искаженными фактами, а также не включают научно-обоснованное документирование и доказательную базу для выработки правовых решений. Отчеты о посещениях стран представителями МККН не заостряют внимание на стереотипах поведения правоохранительных органов, которые ускоряют распространение ВИЧ-инфекции и допускают явные нарушения прав человека. Комитет должен нести ответственность за обеспечение доступа к законным опиоидам на уровне стран; тем не менее Комитет не принял меры для оказанию помощи странам в проведении точной оценки их потребности в заместительной терапии, доказавшей свою эффективность в снижении риска распространения ВИЧ и повышении приверженности к АРВ-терапии.

В своем мандате Комитет подчеркивает важность такого аспекта, как осуществление контроля за наркотиками. Тем не менее в годовом отчете за 2005 г. 18 раз упоминается влияние потребления наркотиков на рост распространения ВИЧ-инфекции в разных странах. Характерно, что этот отчет, как и другие документы МККН, не призывает страны к претворению в жизнь апробированных стратегий снижения распространения ВИЧ среди потребителей наркотиков. Несмотря на то, что решения конвенций ООН по контролю за наркотиками недвусмысленно требуют, чтобы потребителям наркотиков предоставлялось лечение, МККН продолжает хранить молчание в отношении нехватки эффективных программ лечения химических зависимостей и в отношении нарушений прав человека, совершаемых под прикрытием лечения наркозависимости и реабилитации потребителей наркотиков.

Система ООН, в целом, сохраняет приверженность принципам снижения распространения ВИЧ среди потребителей инъекционных наркотиков, охране прав потребителей наркотиков, важности подотчетности перед общественностью и необходимости вовлечения гражданского общества. В этом контексте МККН является исключением, представляя собой закрытую организацию, которая никому не подотчетна, сосредоточена на контроле за наркотиками в ущерб вопросам здравоохранения и призывает национальные правительства делать то же самое.

Члены МККН опровергают или стремятся воспрепятствовать выполнению основанных на фактах рекомендаций, подготовленных другими подразделениями и представителями ООН

- Члены МККН выступают против программ предоставления стерильных шприцев и заместительной терапии опиоидами, несмотря на данные Объединенной программы ООН по ВИЧ/СПИДу (ЮНЭЙДС), Всемирной организации здравоохранения (ВОЗ), Управления ООН по наркотикам и преступности (УНПООН) и самого МККН о том, что эти меры являются эффективным и важным компонентом профилактики ВИЧ-инфекции. В 2002 г. президент МККН использовал в своем заявлении ложную информацию о том, что распространение стерильных шприцев нарушает соглашения конвенций ООН по наркотикам. В 2005 г. в меморандуме, подписанном представителем России в МККН, были приведены многочисленные искажения фактов с целью дискредитация использования метадона для лечения зависимости от опиатов.

- Комитет пытался заставить замолчать представителей ООН, которые поддерживают расширение подходов к профилактике ВИЧ. В 2006 г., например, Стефен Льюис, специальный представитель ООН по вопросам борьбы с ВИЧ-инфекцией в Африке, одобрительно отозвался о полученных в канадском городе Ванкувере данных о снижении риска распространения ВИЧ-инфекции благодаря пункту безопасных инъекций. На следующий день у него в кабинете раздался сердитый звонок из Секретариата МККН, и было обещано, что Комитет обратится с письмом к Генеральному секретарю ООН и попросит выразить Льюису порицание за поддержку «притонов для курильщиков опиума». В письме президент МККН выразил удивление по поводу того, что «сотрудник ООН мог выступить с такими заявлениями», и потребовал, чтобы Льюис публично отказался от своего выступления.

Отчеты МККН восхваляют правительства, которые нарушают права человека

- Делегация Комитета посетила Таиланд в 2004 г., через несколько месяцев после того, как полицией была объявлена «война с наркотиками», в ходе которой специалисты по правам человека зафиксировали факты расправ без суда и следствия, введения квот на количество арестов, использования черных списков и задержания десятков тысяч людей, среди которых многие никогда в жизни не потребляли наркотики. В составленном после посещения страны отчете Комитет не осудил массовые аресты, а совсем наоборот, выразил одобрение правительству Таиланда, которое заявило о намерении провести расследование убийств, и это несмотря на то, что группы правозащитников собрали факты, подтверждающие, что правительство не дало разрешения ни на одно независимое расследование.

- В 2004 г., после того как в Болгарии было введено тюремное заключение за хранение любого количества запрещенных наркотиков, страх ареста привел к резкому увеличению потребления инъекционных наркотиков и совместному использованию шприцев. Представители МККН посетили Болгарию в 2005 г., но в отчете не было даже упоминания о суровом законодательстве в отношении наркотиков или его последствиях, вместо этого было сказано, что национальное законодательство по контролю над наркотиками было «хорошо разработано».

- В 2005 г. в России власти предприняли попытку отменить законодательную реформу, в соответствии с которой были снижены сроки тюремного заключения за хранение очень малых доз наркотиков. Доказано, что огульное назначение больших сроков тюремного заключения привело как к переполнению исправительных учреждений, так и к одному из самых высоких в мире темпов распространения ВИЧ в России. Представители МККН посетили Россию во время обсуждения в стране этой реформы, но в представленном по итогам визита отчете не было упоминания ни о дискуссии, ни об озабоченности тем, что такая политика может оказать негативное влияние на соблюдение прав человека. Вместо этого МККН выразил озабоченность ростом потребления наркотиков в России и призвал к координации и объединению усилий служб, предоставляющих лечение для ВИЧ-инфицированных, и служб, занимающихся лечением наркозависимости.

- С 1990 г. введенный ООН Международный день протеста против злоупотребления и торговли запрещенными наркотиками в Китае отмечается показательными судебными процессами, в которых торговцам наркотиков оглашается смертный приговор, иногда сопровождаемый выкриками из толпы «убить, убить». Десятки были казнены. Несмотря на провозглашаемую поддержку чуткого отношения в политике, МККН не отреагировал критически ни на эту практику, ни на преследования полицией людей, которые стремятся найти стерильное инъекционное оборудование как в Китае, так и любой другой стране, где побывали представители Комитета.

МККН акцентирует внимание на контроле за наркотиками в ущерб вопросам здравоохранения и выражает озабоченность проблемой утечки легальных наркотических средств на черный рынок вместо того, чтобы поощрять принятие научно-обоснованных мер снижения вреда, включая снижение распространения ВИЧ

- Признавая тот факт, что ВОЗ внесла метадон и бупренорфин в Перечень жизненно важных лекарств в 2005 г., Комитет не предпринял никаких видимых усилий для продвижения заместительной терапии (ЗТ) опиоидами в странах с большим количеством потребителей инъекционных наркотиков. Комитет также не сумел подчеркнуть, что ЗТ является существенно важным средством для лечения и помощи ВИЧ-инфицированным. Хотя в последнее время в таких странах, как Украина, Китай, Малайзия и Иран наблюдается тенденция расширения программ ЗТ или/и обмена игл с целью снижения темпов распространения ВИЧ, отчеты МККН не содержат ни оценки, ни удовлетворения этими фактами. Более того, Комитет выразил озабоченность утечкой метадона и бупренорфина на черный рынок и призвал ВОЗ проводить агитацию за ужесточение контроля за использованием данных препаратов.

МККН публикует свою интерпретацию законодательства и связанных со снижением вреда высказываний, не имея достаточной экспертизы в области международного права и политики в отношении ВИЧ

- Согласно опубликованным биографическим справкам, ни один из 13 членов МККН не имеет формального образования или подготовки в области международного права, несмотря на то, что этот фактор является важным требованием при толковании положений международных

договоров. В вопросах, связанных с заместительной терапией, обменом игл, пунктами безопасных инъекций, высказывания членов МККН противоречат данным исследований, проведенных их собственными консультантами по правовым вопросам и национальными экспертами.

- Несмотря на центральное значение потребления наркотиков в передаче ВИЧ-инфекции, ни один из членов Комитета не имеет публикаций в рецензируемых журналах по вопросам ВИЧ/СПИДа, и лишь несколько человек упоминают наличие какого-либо опыта в лечении или профилактике ВИЧ в своих биографических справках.

Комитет работает в атмосфере секретности и не имеет механизма для соблюдения подотчетности

- Заседания МККН закрыты для наблюдателей, протоколы заседаний не предоставляются.
- Члены МККН используют свое положение в Комитете, выступая с утверждениями, основанными на искаженных фактах, однако не существует открытого для общественности механизма, который бы позволил государствам-участникам или общественным организациям опротестовать эти утверждения, попросить разъяснения и уточнения или предложить поправки.
- Источники приводятся в отчетах МККН избирательно и непоследовательно.
- МККН не оглашает заранее свою программу поездок в страны, не созывает общественные слушания, не предоставляет никаких других возможностей поделиться мнением.
- Несмотря на призыв Генерального секретаря ООН к большей прозрачности и укреплению взаимодействия с гражданским обществом в сети ООН, на вебсайте МККН не приводится информации о бюджете или сотрудниках Комитета. Секретариат МККН, оплата содержания которого производится за счет ООН, не отвечает на запросы неправительственных организаций или сообществ, затронутых проблемой ВИЧ.

Рекомендации

Для улучшения подотчетности, лучшего реагирования на проблему эпидемии ВИЧ и соответствия своему мандату - оценке выполнения конвенций ООН по контролю за наркотиками - МККН должен измениться.

- МККН должен регулярно оценивать спрос и адекватность проводимого в странах лечения химических зависимостей. Он должен предоставлять техническую помощь странам для проведения точной оценки их потребности в заместительной терапии опиоидами. Комитет должен поддерживать государства, которые стремятся увеличить масштабы применения этого лечения, и поощрять к поиску безопасных и эффективных способов внедрения заместительной терапии государства, которые только еще рассматривают вопросы применения этого спасающего жизни лечения.
- В своих наблюдениях за практикой потребления наркотиков и охраной здоровья МККН должен ссылаться на научно-обоснованные данные, а

также приводить юридическое обоснование своей интерпретации законодательства. Свои ежегодные отчеты Комитет должен снабжать ссылками на источники информации и предоставлять возможность государствам-членам ООН и группам гражданского общества предлагать изменения или дополнительную информацию.

- МККН должен предоставлять большую возможность обмена мнениями с государствами-членами ООН, имеющими соответствующий мандат организациями ООН, гражданским обществом и экспертами в области ВИЧ/СПИДа. МККН должен включать возможности для вовлечения этих групп в свою работу, планируя свои страновые миссии.

- Всемирная организация здравоохранения, Экономический и социальный Совет ООН и государства-члены ООН должны стремиться к тому, чтобы в МККН входили люди, имеющие экспертизу в области международного права и политики по ВИЧ/СПИДу.

- МККН должен сформулировать, а ЭКОСОК оценить основные рекомендации для процедуры внесения поправок в случаях, когда отдельные члены МККН выступают от имени всего Комитета и оперируют искаженными фактами.

- Генеральный секретарь ООН должен поручить независимой организации провести оценку работы МККН, включая научную оценку высказываний Комитета по вопросам здравоохранения, и рассмотреть факторы независимости и опыта членов Комитета, уделяя особое внимание вопросам ВИЧ, международного права и прав человека.

V. Выводы и рекомендации

В различных областях МККН проявил лидерство и дальновидность, которые выходят за рамки контроля над наркотиками и затрагивают другой важнейший принцип конвенций ООН по проблеме наркотиков, а именно - облегчение человеческих страданий. МККН приложил усилия к предоставлению адекватного наличия контролируемых лекарственных препаратов для облегчения боли пациентам, страдающим от раковых заболеваний и ВИЧ. В частности, важность лидерства Комитета проявилась в сотрудничестве с другими организациями ООН и налаживании диалога с Секретариатом в Вене и разными странами. В дополнении, в обсуждениях программ предоставления альтернативных источников дохода фермерам, которые выращивают запрещенные культуры, Комитет также призывал к использованию таких мер, как «полное участие фермеров, их семей и местной общины» в определении путей решения проблем, и указывал на важность признания ситуаций, при которых «незащищенность, безысходность и отсутствие каких-либо прав и возможностей повышают опасность злоупотребления наркотиками и наркомании».[122]

МККН явно осознает, что изменение в понимании последствий потребления наркотиков требует изменений в расстановке акцентов. В вопроснике МККН «Оценка осуществления международных договоров о контроле над наркотиками», посвященном изменениям, произошедшим после Специальной сессии Ассамблеи ООН 1998 г. по проблемам борьбы с наркотиками, например, используется термин «наркозависимые» вместо термина «люди, злоупотребляющие наркотиками». Это свидетельствует о внимании к вопросам лечения химических зависимостей и доступа к услугам реабилитационных учреждений и показывает стремление МККН к наличию в странах мер по снижению вреда, связанного с наркотиками.[123] В различных ситуациях

Комитет признавал сложность проблемы наркозависимости и ее связи с социальными, культурными и экономическими факторами, предлагая при этом способы вовлечения общественности и смягчения наказания с целью возможности повторной интеграции в общество людей, потребляющих наркотики.[124]

Однако Комитет пока не признал, что те же подходы, которые применимы для людей, выращивающих запрещенные культуры, применимы и для людей, потребляющих наркотики: эти люди имеют право на участие в принятии решений и разработке программ, которые влияют на их жизнь. Такое участие повышает эффективность усилий, которые направлены на устранение проблем, связанных с запрещенными наркотиками. Непризнание данного факта особенно сказывается на проблеме ВИЧ/СПИДа, в то время как важность участия сообществ, затронутых эпидемией, и внимание к правам человека постоянно подчеркиваются организациями ООН и национальными правительствами.

К сожалению, как показано на этих страницах, МККН остается пока глухим к голосу здравого смысла по многим критически важным вопросам профилактики и лечения ВИЧ.

Рекомендации

Для того, чтобы МККН работал в одном ключе с другими международными организациями в создании серьезного и эффективного ответа на проблему ВИЧ/СПИДа, необходимо предпринять перечисленные ниже меры.

Уделять больше внимания наличию и качеству лечения химических зависимостей: Комитет должен регулярно оценивать и сообщать сведения о наличии и качестве предлагаемого лечения химических зависимостей, основываясь на лучшей международной практике и конвенциях ООН по борьбе с наркотиками. В странах, где распространение ВИЧ-инфекции в большой мере обусловлено потреблением инъекционных опиоидов, такая оценка должна включать степень координации программ лечения ВИЧ/СПИДа и наркозависимости, доступность заместительной терапии опиоидами, определение потребностей в таком лечении. Если ЗТ является незаконной или недоступной, МККН должен проводить работу, как с органами здравоохранения, так и с органами, осуществляющими контроль за наркотиками, чтобы преодолеть препятствия для эффективного применения ЗТ. Комитет должен подчеркивать совместимость ЗТ с положениями конвенций ООН по борьбе с наркотиками в таких странах, как Россия, где власти и члены МККН утверждают, что метадон подпадает под запрещенные вещества на основании конвенций ООН. Комитет должен рекомендовать техническую или финансовую помощь с целью содействия странам в предоставлении незапрещенных опиоидов в количествах, необходимых для всех видов использования в медицинских целях, включая лечение зависимостей. Показатели успешной деятельности МККН должны включать механизмы мониторинга и обеспечения доступа к лечению химических зависимостей, как, например, количество направленных в правительственные учреждения писем, в центре внимания которых - доступ к метадону и другим заместителям опиоидов как компонент национальных программ по ВИЧ/СПИДу; а также количество выступлений в печати, где подчеркивается озабоченность МККН нарушениями прав человека, совершаемых во имя лечения и реабилитации потребителей наркотиков.

Более тщательно документировать правовые заключения и заявления об эффективности стратегий снижения вреда: отсутствие ссылок на опубликованные материалы в ежегодных отчетах и заявлениях МККН подрывает доверие к Комитету.

МККН должен документировать результаты своей работы так же, как это делается в технических отчетах ВОЗ. Наблюдения, сделанные в ходе страновых визитов, должны именно так и обозначаться, и замечания о состоянии мер здравоохранения, которые стали предметом рецензированного научного исследования, должны снабжаться ссылками на это исследование. Отчеты МККН должны включать обзор результатов Меморандума 2002 года Правового отдела Программы ООН по контролю над наркотиками о совместимости пунктов безопасных инъекций и программ предоставления стерильных шприцев с международными конвенциями. Комитет должен также проводить анализ и предоставлять ссылки на другие опубликованные аналитические и исследовательские материалы об эффективности использования таких пунктов и программ стерильных шприцев в целях направления их клиентов на лечение наркозависимости и снижения риска распространения ВИЧ-инфекции без увеличения потребления запрещенных наркотиков.

Увеличить прозрачность и подотчетность, в частности, через привлечение государств-членов ООН и гражданского общества: ежегодный отчет МККН задает тон обсуждениям в Комиссии по наркотическим веществам (CND) и широко освещается в СМИ. Государства-члены ООН и гражданское общество должны иметь возможность регулярно обмениваться мнениями с Комитетом и предлагать отзывы на приведенные в отчете данные. Руководящие органы организаций ООН и таких крупных организаций, как Глобальный фонд по борьбе со СПИДом, туберкулезом и малярией учредили официальные механизмы для участия гражданского общества в обсуждении их деятельности. МККН должен сделать то же самое, хотя бы для своей собственной информации. МККН должен заранее оповещать о страновых визитах и отводить время для обмена мнениями с лицами, ответственными за формирование политики и стратегий в отношение наркотиков, и организациями гражданского общества, представляющими интересы пользователей наркотиков и других людей, на жизнь которых повлияла эпидемия ВИЧ/СПИДа. Было бы полезно, если бы Комитет обнародовал обоснование своей политики проведения закрытых совещаний, дискуссий и корреспонденции. Такой анализ должен включать оценку возможности проведения неофициальных сессий при участии ООН, национальных правительств и неправительственных организаций.

Приложить больше усилий к тому, чтобы в Комитет были включены люди с экспертизой в области ВИЧ: учитывая важность деятельности МККН для борьбы с глобальной эпидемией ВИЧ, среди членов Комитета должны быть люди, имеющие экспертизу в области ВИЧ-инфекции. Всемирная организация здравоохранения должна гарантировать, что среди пяти ее кандидатов хотя бы трое имеют значительную и убедительную экспертизу в наркополитике и области ВИЧ/СПИДа. ЭКОСОК должен направить просьбу в адрес государств-членов ООН рассматривать наличие экспертизы в области ВИЧ/СПИДа при выдвижении кандидатов в МККН. ЭКОСОК также должен поощрять государства-члены ООН к включению в число кандидатов людей с экспертизой в области международного права.

Принципы независимости членов Комитета: МККН должен сформулировать, а ЭКОСОК оценить принципы независимости членов Комитета, чтобы для общественности было понятно, когда член Комитета выражает мнение от лица всего Комитета, а когда от себя лично, и как можно внести поправку в неверно представленные факты.

Независимая оценка деятельности МККН: Генеральный Секретарь ООН должен поручить независимой организации провести оценку работы МККН.

В ходе такой оценки должны быть проведены консультации со всеми организациями ООН - соучредителями ЮНЭЙДС, экспертами и организациями, работающими над проблемами профилактики ВИЧ-инфекции среди потребителей наркотиков. Оценка должна включить такой вопрос, как влияние высказанных членами МККН мнений и замечаний на национальную политику, а также влияние работы МККН в целом на политику в отношении ВИЧ-инфекции. Кроме того, должна быть дана оценка независимости и объективности членов МККН и критериям, которыми руководствуются государства-члены ООН и ВОЗ при выдвижении кандидатур в члены Комитета. Контролирующая функция, возложенная на Генерального секретаря ООН в решениях Единой Конвенции ООН о наркотических средствах 1961 г., даёт ему такое право.

[122] Доклад Международного комитета по контролю над наркотиками за 2005 г., параграф 23 (g)

[123] Международный комитет по контролю над наркотиками. Вопросник: Оценка осуществления международных договоров о контроле над наркотиками, 2007 год. Вопросник можно найти по следующему адресу: http://www.incb.org/pdf/questionnaire/Questionnaire_Rus.doc

[124] См., например, Доклад Международного комитета по контролю над наркотиками за 2004 г., параграф 2 и Доклад Международного комитета по контролю над наркотиками за 2003 г., параграф 59
