# Supplementary material for: Closed to reason: time for accountability for the International Narcotic Control Board
Source: Harm Reduct J. 2007 May 8;4:13. doi: 10.1186/1477-7517-4-13 (PMC1871577; doi:10.1186/1477-7517-4-13)
Supplement: Additional file 3 — "Closed to Reason": Time for Accountability for the International Narcotic Control Board. Russian translation of abstract of the above editorial. [file 1477-7517-4-13-S3.pdf]

Резюме

В течение более двух десятков лет Международный совет по контролю над наркотиками (INCB), пытался остановить развитие программ снижения вреда и профилактики ВИЧ. Такая позиция Совета исходит из полного непонимания своей ответственности и природы самой наркомании, т.е. вопрос общественного здравоохранения и клинического ухода, стал по решению Совета уголовным вопросом. Новым центром внимания Совета стали усилия, направленные на отказ от использования учреждений контролируемого потребления наркотиков для снижения заболеваемости и смертности людей, употребляющих инъекционные наркотики. Члены Совета выбрали отдельные страны и пытаются заставить их отказаться от таких программ под знаменем Организации объединенных наций (фальшиво) прикрываясь международными договорами. Их неослабевающие и неоправданные придирки к странам, подписавшимся под международными договорами, учредившими INCB, являются не только неоправданными, но и противоречат основным целям самого Совета, которые состоят в обеспечении соответствующими медикаментами и безопасного применения контролируемых веществ. Навязчивая идея INCB о том, что пристрастие к инъекционным наркотикам является преступлением, попадает в медицинское поле зрения и руководящие установки ООН и единодушно одобряется Генеральной Ассамблеей ООН.

Недавней мишенью INCB стало единственное в Северной Америке учреждение контролируемого потребления наркотиков, находящееся в старой центральной части Ванкувера, Канада. Использование полномочий своего ведомства для вмешательства в дела общественного здравоохранения отдельных стран не имеет ни медицинского, ни научного, ни юридического обоснования. Но самое главное, что это вопрос жизни и смерти для наиболее маргинализированной группы граждан. Эмпирические свидетельства продолжают указывать на то, что неослабевающий рост пандемии СПИДа происходит, в основном, в результате инъекционного потребления наркотиков, и вмешательство INCB неизбежно приведет к новым смертным случаям в результате ВИЧ инфекций и передозировок наркотиков, которые можно было бы предупредить.

Мы рады привлечь внимание наших читателей к последнему отчету, подготовленному Канадской правовой сетью ВИЧ/СПИДа и Международной программой развития снижения вреда (IHRD) совместно с бывшим Послом ООН для особых поручений в Африке, уважаемым канадским политиком, Стефеном Льюисом. Мы прилагаем полную версию отчета «Закрыто для здравого смысла: Международный Совет по контролю над наркотиками и ВИЧ/СПИД». Отчет четко дает понять, что сейчас настало время ввести немного ответственности и здравого смысла в INCB.
