# Supplementary material for: Closed to reason: time for accountability for the International Narcotic Control Board
Source: Harm Reduct J. 2007 May 8;4:13. doi: 10.1186/1477-7517-4-13 (PMC1871577; doi:10.1186/1477-7517-4-13)
Supplement: Additional file 4 — "Closed to Reason": Time for accountability for the International Narcotic Control Board. Chinese translation of abstract of the above editorial. [file 1477-7517-4-13-S4.pdf]

理智的封閉
是時候為國際毒品管理局(INTERNATIONAL NARCOTIC CONTROL BOARD)建立問責制度

撮要
過去二十年來，國際毒品管理局(International Narcotic Control Board (INCB))企圖制止「減少傷害」與及它的愛滋病預防計劃，這一姿態是建基於對他們責任與及毒品癖癮的根本誤解；哪就是毒品癖癮是一種公共醫療與及醫護診斷的問題，卻被判定為犯罪行為。該局最近的焦點是拒絕讓安全注射設施減少注射吸毒人士的染病率與及死亡率。他們針對個別國家，欺壓這些國家撤銷這些計劃，打著(虛假的)聯合國旗幟，與及以國際條約為藉口。他們竭盡所能而又毫無根據的糾纏建立INCB的國際條約簽署國，不單只毫無理據，而且冒犯了該局的其中一個核心目標：確保受管制藥物能夠得到充足供應，予醫療使用，與及安全地使用。INCB著魔一樣的把注射毒品當作犯罪行為，與世界衛生組織(World Health Organization)與及聯合國大會所秉承而且普世接受的原則有所衝突。

INCB的最新目標是北美洲最大型的受監察注射設施，設於加拿大溫哥華的Insite。使用他們機關的權力介入個別個別國家的公共醫療事務，是沒有醫療、科學、與及法理根據的。但最為重要的是，對於這些最為邊緣化的公民來說，是一件關乎生死的事。積累下來的證據顯示，愛滋病不斷擴散有很大比例由毒品注射引起。INCB的干擾無可避免帶來更多死亡，不論是因為愛滋病還是吸食過量毒品，均是可以制止的。

我們很高興可以向讀者推薦，由加拿大愛滋病法律網絡(Canadian HIV/AIDS Legal Network)與及國際減少傷害計劃(International Harm Reduction Development Program (IHRD))與前聯合國非洲愛滋病特使，受人尊重的加拿大政治家Stephen Lewis所共同撰寫的一份報告。附上的是整份報告，理智的封閉- 是時候讓國際毒品管理局建立問責制度(Closed to Reason: The International Narcotics Control Board and HIV/AIDS)。正如報告所說，是時候為INCB注入一點問責與理智。
